# Immune function and dysfunction are determined by lymphoid tissue efficacy

**DOI:** 10.1242/dmm.049256

**Published:** 2022-01-24

**Authors:** Spyridon Makris, Charlotte M. de Winde, Harry L. Horsnell, Jesús A. Cantoral-Rebordinos, Rachel E. Finlay, Sophie E. Acton

**Affiliations:** 1 Stromal Immunology Group, MRC Laboratory for Molecular Cell Biology, University College London, Gower Street, London WC1E 6BT, UK; 2 Department for Molecular Cell Biology and Immunology, Amsterdam UMC, location VUmc, De Boelelaan 1108, 1081 HZ Amsterdam, Netherlands; 3 Division of Immunology, Immunity to Infection and Respiratory Medicine, School of Biological Sciences, The University of Manchester, Manchester M13 9PL, UK

**Keywords:** Fibroblastic reticular cells, Homeostasis, Lymphoid tissue, Stromal cells

## Abstract

Lymphoid tissue returns to a steady state once each immune response is resolved, and although this occurs multiple times throughout life, its structural integrity and functionality remain unaffected. Stromal cells orchestrate cellular interactions within lymphoid tissue, and any changes to the microenvironment can have detrimental outcomes and drive disease. A breakdown in lymphoid tissue homeostasis can lead to a loss of tissue structure and function that can cause aberrant immune responses. This Review highlights recent advances in our understanding of lymphoid tissue function and remodelling in adaptive immunity and in disease states. We discuss the functional role of lymphoid tissue in disease progression and explore the changes to lymphoid tissue structure and function driven by infection, chronic inflammatory conditions and cancer. Understanding the role of lymphoid tissues in immune responses to a wide range of pathologies allows us to take a fuller systemic view of disease progression.

## Abstract (precis)

**Summary:** Lymphoid tissue fitness can determine disease severity and affect outcome by altering the course of immune responses. We discuss how lymphoid tissue function can impact disease progression during infections, chronic inflammatory conditions and cancer.

## Introduction

A state of health is maintained through a fine balance between immune responses against a pathogen and the ability to return to steady state while minimising tissue damage. Leukocytes constantly interact with stromal cells in primary and secondary lymphoid organs and are maintained in a response-ready state. Mature lymphocytes develop in primary lymphoid organs, the thymus and bone marrow, and require survival signals and growth factors within secondary lymphoid organs, including lymph nodes, spleen, tonsils and Peyer's patches, to mount adaptive immune responses (Ruddle and Akirav, 2009). Lymph nodes originate during embryonic development and are not fully established until after birth.

A lymph node anlage is initiated at embryonic day 12.5-13.5 in mice and is composed of both haematopoietic lymphoid tissue inducer (LTi) cells and lymphoid tissue organiser (LTo) cells, the stromal precursors. Multiple mouse models show that LTi cells are integral for the formation of lymph nodes (Fletcher et al., 2015; Krishnamurty and Turley, 2020). Within the foetal liver, common lymphoid progenitors differentiate into α-lymphoid precursor cells that preferentially differentiate into LTi precursors but acquire their full functional phenotype only after they have reached peripheral tissues (Simic et al., 2020). During lymph node organogenesis, receptor activator of NF-κB (RANK; also known as TNFRSF11A)-mediated lymphatic endothelial cells (LECs) control the retention of LTi cells in the embryonic lymph node analgen (Onder et al., 2017). LTi progenitors arise from embryonic hemogenic endothelium, which are replaced by haematopoietic stem cell-derived LTi cells in adults (Simic et al., 2020). LTi cells (CD45^+^CD3^−^RORγT^+^; also known as PTPRC^+^CD3^−^RORC^+^) in adult tissues are part of the innate lymphoid cell (ILC) family (see Glossary, Box 1), and their recruitment to lymph nodes is driven by chemokines such as CXCL13 (Brendolan and Caamaño, 2012; Eberl et al., 2003; Krishnamurty and Turley, 2020; Luther et al., 2003). LTi cells express lymphotoxin-α1β2 (Box 1), RANK, RANK-ligand (RANKL; also known as TNFSF11), IL-7R, integrin α4β7, and the chemokine receptors CXCR5 and CXCR7 (also known as ACKR3) (Cupedo et al., 2004; Krishnamurty and Turley, 2020; Mueller and Hess, 2012), all of which contribute to stromal recruitment and development of the lymphoid tissue niche. LTo stromal cells are CD45^−^LTβR^+^ and can arise from multiple origins. Fate mapping in mice suggests that mesenchymal LTo cells arise from fibroblast activation protein-α (FAP)^+^ embryonic precursors (Denton et al., 2019). However, LTo stromal precursors have also been shown to be nestin^+^ and to contribute to both mesenchymal and endothelial stromal populations within developing lymph nodes (Koning et al., 2016). Interaction between LTo cells and infiltrating LTi cells induces the secretion of lymphotoxin, IL-7, RANKL, and chemokines CCL19, CCL21 and CXCL13, attracting and retaining lymphocytes in the developing tissue. LTo stromal precursors also upregulate the adhesion molecules VCAM-1, ICAM-1 and MAdCAM-1 (Bénézech et al., 2010; Fletcher et al., 2015), augmenting further recruitment and survival of LTi cells in the lymph node anlagen. The close interaction between LTo and LTi cells creates a positive feedback loop to increase cellularity during development and initiate tissue organisation and lymphocyte compartmentalisation in the developing lymph node.

Mature lymph nodes are compartmentalised into specialised microenvironments that ensure optimal adaptive immune responses (Girard et al., 2012). Mesenchymal and endothelial stroma constitute less than 2% of the cell population in the lymph node, yet regulate the structure and function of the tissue. There are four major populations of stromal cells: fibroblastic reticular cells (FRCs), follicular dendritic cells (FDCs), blood endothelial cells (BECs) and LECs. Single-cell transcriptomics studies have identified divergent stromal cell subphenotypes linked to distinct niches within the lymph node (Brulois et al., 2020; Fujimoto et al., 2020; Rodda et al., 2018; Xiang et al., 2020), showing that the precise location of stromal cells further specialises their phenotypes (Box 2). Maturing stromal cells generate chemoattractant gradients that support the separation of lymphocyte niches (Bénézech et al., 2010). The fibroblastic stroma composes an interconnected cellular network, termed the FRC network, which confines a bundled extracellular matrix (ECM) through which lymph fluid from peripheral tissues is filtered (Acton et al., 2021). The unique structure of lymph nodes therefore confers effective communication between peripheral tissues, leukocytes and the stromal cell architecture. The robust homeostatic state of lymph nodes facilitates effective protective adaptive immune responses repeatedly throughout our long lifespans, balancing inflammatory signalling with tolerance of self-antigens. If this homeostatic state is perturbed, the lymph node loses its architecture and function, with severe consequences for immunity.

Beyond secondary lymphoid organs, such as lymph nodes, organised aggregations of lymphocytes can spontaneously arise in a range of inflammatory conditions, forming tertiary lymphoid structures (TLSs) (Pipi et al., 2018). The role of TLSs in disease progression is complex. In autoimmune disorders, TLSs are associated with production of the autoantibodies that drive disease progression; conversely, TLS formation correlates with good patient outcomes for many cancer types, suggesting that TLSs potentially promote anti-tumour immune responses (Pipi et al., 2018). Similarly to lymph nodes, TLSs can exhibit segregated B- and T-cell zones (Da Graça et al., 2021; Rodriguez et al., 2021), and are supported by stromal cells including FDCs, endothelial cells and fibroblasts (Da Graça et al., 2021; Mitsdoerffer and Peters, 2016). The mechanisms of TLS initiation are not fully understood, but are likely to be driven by infiltrating cells and context-dependent inflammatory cues (Mitsdoerffer and Peters, 2016; Rodriguez et al., 2021). Similarly to lymph node development, FAP^+^ stromal cells can initiate lymphatic aggregates in response to inflammation (Denton et al., 2019), requiring the presence of lymphotoxin for TLS formation and maintenance (Mitsdoerffer and Peters, 2016). TLSs maintain the minimal structural and functional parallels with secondary lymphoid tissues.

In this Review, we discuss the role of secondary and tertiary lymphoid tissues in homeostasis and disease states, and the impact of lymphoid tissue function on disease progression.

### Stromal cell control of immune function

Both blood and lymphatic endothelial cell populations regulate leukocyte traffic through the lymph node (Ager, 2017; Herzog et al., 2013; Jalkanen and Salmi, 2020). There are six subpopulations of LECs, each with distinct roles in accommodating leukocyte migration and niche maintenance via secretion of growth factors and chemokines (Jalkanen and Salmi, 2020). During development, LECs produce CXCL13 to recruit LTi cells (Bovay et al., 2018). Podoplanin (PDPN) expression on LECs is important for homing migrating CLEC-2^+^ dendritic cells (DCs) (Acton et al., 2012; Krishnamurty and Turley, 2020). Floor and ceiling LECs, located in the subcapsular sinus of a lymph node, create a chemokine gradient by expressing CCL21/CCL19 and the scavenger chemokine receptor CCRL1 (also known as ACKR4), respectively, which drives directional migration of CCR7^+^ leukocytes toward the cortical regions (Luther et al., 2003; Ulvmar et al., 2014). Specialised blood vessels, the high endothelial vessels (HEVs), also support lymphocyte trafficking into and out of the lymph node (Ager, 2017; Nourshargh et al., 2010), and HEV neogenesis has also been observed in TLSs (Jones et al., 2018). To balance lymphocyte trafficking and maintain vascular integrity, lymph node HEVs are supported by perivascular PDPN^+^ FRCs that bind to and activate CLEC-2^+^ platelets. The activated platelets release sphingosine-1-phosphate, reinforcing cell–cell junctions via VE-cadherin (Herzog et al., 2013).

FRCs are the most abundant stromal cell in the lymph node, forming a connected cellular network spanning the entire tissue and primarily supporting the paracortical region, also referred to as the T-cell zone. FRCs express platelet-derived growth factor receptor alpha (PDGFRα) and the membrane glycoprotein PDPN. The ontogeny of FRCs is not fully characterised and they may arise from different sources during development and in adults (Koning et al., 2016). FRCs express the newly determined universal fibroblast marker dermatopontin, but their phenotype is believed to be imprinted by their tissue of residence (Buechler et al., 2021). FRCs can derive from mesenchymal precursors migrating from local adipose tissue and from the differentiation and proliferation of marginal reticular cells from within the lymph node (Bénézech et al., 2012; Gil-Ortega et al., 2013; Katakai et al., 2008). The Hippo signalling pathway controls the growth and development of various tissues (Cho et al., 2019; Yu et al., 2015), and its dysregulation during FRC development and maturation suggests that YAP/TAZ (Box 1; also known as YAP1/ TAFAZZIN) are critical regulators for FRC commitment and maturation (Choi et al., 2020). Specifically, YAP/TAZ deficiency impairs FRC development, whereas their hyperactivation promotes myofibroblastic conversion and tissue fibrosis (Choi et al., 2020). These changes to FRC phenotype are detrimental to lymph node function during immune responses.

**Fig. 1.**
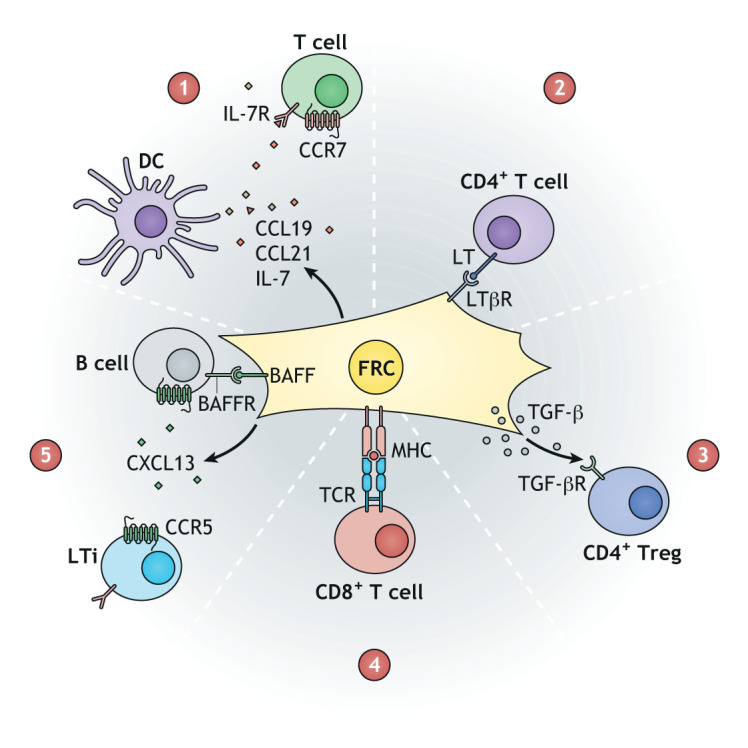
**Fibroblastic reticular cells (FRCs) are central in maintaining the lymph node niche.** (1) Lymph node FRCs produce the chemokines CCL19 and CCL21 to recruit dendritic cells (DCs) and T cells that express CCR7. Secretion of IL-7 by FRCs drives the survival of T cells within the lymph node microenvironment. (2) CD4^+^ T cells express lymphotoxin (LT), which interacts with the LTβ receptor (LTβR) on FRCs and thus provides the survival factor for FRCs. (3,4) FRCs have immunoregulatory roles: they can activate regulatory T cells (Tregs) through the secretion of TGF-β (3) or induce peripheral tolerance of CD8^+^ T cells through the interaction between major histocompatibility complex (MHC) and T-cell receptor (TCR) (4). (5) FRCs can produce CXCL13, which can recruit lymphoid tissue inducers (LTi) and B cells. B-cell survival is also driven by the interaction between the surface B-cell activating factor (BAFF) on FRCs and the BAFF receptor (BAFFR) on B cells. TGF-βR, TGF-β receptor.

FRCs maintain structural integrity during the rapid tissue remodelling of lymph node expansion and support leukocyte trafficking for efficient antigen presentation. Communication between the FRC network and immune cells instructs stromal cytoskeletal remodelling to accommodate the dynamic network remodelling required for adaptive immune responses (Acton et al., 2014; Astarita et al., 2015; Fletcher et al., 2015). The PDPN expressed on the surface of the FRC network also acts as a ligand promoting DC migration, supporting their search for cognate T cells (Acton et al., 2012). FRCs in the T-cell zone secrete CCL19, CCL21 and IL-7, which attracts and retains CCR7^+^IL7R^+^ naïve T cells (Fig. 1) (Förster et al., 1999; Gunn et al., 1999; Link et al., 2007). FRCs function as immunomodulators by secreting nitric oxide, COX-2 (also known as PTGS2) and the immunosuppressive metabolic enzyme IDO (Box 1; also known as IDO1) (Brown et al., 2019; Siegert et al., 2011), and can directly present self-antigens to promote immune tolerance (Fletcher et al., 2015; Lee et al., 2007). A subset of Gremlin^+^ FRCs located between the T- and B-cell zones is associated with DC maintenance (Kapoor et al., 2021), while CCL19^+^ FRCs can sense type I Interferons (IFNs) during viral infections, support the myeloid composition and reduce CD8^+^ T-cell exhaustion (Perez-Shibayama et al., 2019). FRCs nearer to the B-cell follicles also drive the survival and attraction of B lymphocytes by secreting B-cell activating factor (BAFF; also known as TNFSF13B), IL-6 and CXCL13 (Fig. 1) (Cremasco et al., 2014; Huang et al., 2018; Mionnet et al., 2013). Indeed, FRC depletion in CCL19-Cre × iDTR mice caused a loss of T cells and of conventional and migrating DCs, but also affected B-cell function and humoral immunity (Cremasco et al., 2014). These findings highlight the numerous immunomodulatory functions of the FRC network in coordinating and maintaining a favourable niche for immune cells during homeostasis and immune responses.

Lymph nodes constantly filter draining lymph via a conduit network of aligned ECM fibrils ensheathed by FRCs (Kaldjian et al., 2001; Sixt et al., 2005). The containment of draining lymph by the FRC network allows for the controlled sensing of soluble mediators from peripheral tissues, but also provides a route to secrete antibodies and other lymphoid tissue-derived molecules out of the lymph node (Acton et al., 2021; Thierry et al., 2018). The ECM can also retain chemokines such as CXCL13 to preserve B cells within the follicular region by creating a chemokine gradient (Cosgrove et al., 2020). FRCs deposit the ECM basolaterally in a process controlled by LL5-β (also known as PHLDB2) and CLEC-2 (also known as CLEC1B) expressed by DCs (Martinez et al., 2019). Taken together, lymph node homeostasis is a delicate balance driven by the stromal microenvironment, particularly FRCs, supporting all aspects of adaptive immunity.

**Box 1. Glossary**

**Alarmin:** a type of danger-associated molecular pattern released from necrotic tissue. Implicated in a range of processes, including homeostasis, autoimmunity and cancer.

**Graft versus host disease (GVHD):** a breakdown of homeostasis after organ transplantation that is initially driven by donor T cells reacting against host tissues. The most common target tissues are the skin, intestine and secondary lymphoid organs, such as the lymph nodes and spleen.

**iFABPtOVA mouse model:** a murine model of autoimmunity developed by Vezys et al. (2000), where ovalbumin (OVA) is presented as a self-antigen by epithelial cells. These epithelial cells can be targeted by OTI CD8^+^ T cells to replicate a breakdown in tolerance, allowing researchers to study the underlying mechanisms.

**Indoleamine 2,3-dioxygenase (IDO):** an immunoregulator enzyme that controls tryptophan levels. Cancer cells alter their microenvironment by upregulating IDO, depleting tryptophan, which allows their survival.

**Innate lymphoid cell (ILC):** a type of lymphoid cell that lacks the receptors that recognise antigens. ILCs are involved in lymphoid tissue development and homeostasis and are also required for innate immune responses.

**Kawasaki syndrome:** an acute inflammatory condition of medium/small blood vessels in children under 5 years of age. The mechanisms that initiate and drive the syndrome are not well understood.

**Lymphotoxin:** a member of the tumour necrosis factor cytokine superfamily that is expressed by T lymphocytes. It provides a survival signal for fibroblastic reticular cells (FRCs) by binding to the LTβ receptor. During development it is also expressed by lymphoid tissue inducer (LTi) cells, which drive the survival of lymphoid tissue organiser (LTo) cells.

**Macrophage activation syndrome:** a syndrome associated with prolonged activation of macrophages. Symptoms include cytopenia, organ dysfunction and coagulopathy (Wang et al., 2015).

**Notch signalling:** an evolutionarily conserved mechanism of cell–cell communication that mediates cellular proliferation, cell fate specification, and maintenance of stem and progenitor cells. The four Notch receptors (Notch1-4) interact with Delta-like (DLL)1/3/4 or Jagged (JAG)1/2 ligands. During GVHD, DLL1/4 ligands on the host cells interact with Notch1/2 receptors on donor T cells.

**OVA.CCL19.DTR.CRE^+/−^ model:** a murine model derived from crossing Ccl19-Cre.DTR mice, in which CCL19^+^ FRCs are depleted following diphtheria toxin treatment, with iFABtOVA mice. This model showed that FRCs are required for peripheral tolerance of auto-reactive CD8^+^ T cells (Dertschnig et al., 2020).

**Peripheral tolerance:** the ability of immune cells not to mount an immune response against self-antigens expressed by host tissue. Stromal cells can remove self-reacting lymphocytes in the thymus but also dampen them in secondary lymphoid organs.

**T-helper (Th) cells:** subsets of CD4^+^ T lymphocytes characterised by the cytokines that they secrete. The Th cell family includes Th1, Th2, Th17, Th9, Th22, Tfh and Tregs. Th cells can skew immune responses and have multiple roles, from supporting the local niche, clearing invading pathogens to dampening immune responses.

**YAP/TAZ:** YAP and TAZ are transcriptional co-activators that are part of the Hippo signalling pathway. They can sense mechanical forces in cells and integrate signals from cell junctions, cell polarity and soluble extracellular ligands, and are important drivers of FRC commitment and maturation.

**Box 2. Technical advances in lymphoid tissue characterisation**

To observe three-dimensional (3D) networks of lymph node stromal cells, single-cell, dynamic and temporal images of whole tissues are required (Barbazan and Vignjevic, 2019; Fletcher et al., 2015; Perez-Shibayama et al., 2019). Intravital two-photon microscopy is the standard for imaging deeper into tissues, requiring non-destructive sectioning (Bajénoff et al., 2006; Chauveau et al., 2020; Miller et al., 2002). However, traditional tissue preparations mean that light scattering inhibits deeper imaging. Optical projection tomography and light-sheet fluorescence microscopy have achieved greater macroscopic views but fail to reach subcellular resolutions (Santi, 2011).

Tissue-clarifying techniques such as CLARITY (Chung and Deisseroth, 2013), CUBIC (Susaki et al., 2014) and PACT (Yang et al., 2014) have been developed. PACT techniques identify highly connected networks of cells and reveal conduit structures in the lymph nodes (Martinez et al., 2019; Yang et al., 2014). We are currently able to achieve 3D imaging of tissues, but are unable to visualise single-cell events over larger timespans and at high temporal resolution. Large-volume, high-resolution time-lapsed intravital imaging holds promise for addressing the temporal and spatial challenges. This approach stitches together high-resolution and low-magnification intravital images in surgically immobilised tissues (Entenberg et al., 2017). These methods could be combined with faster temporal resolution imaging, using methods from neuroscience fields (Cui et al., 2020; Wu et al., 2020), to improve our spatial and temporal understanding of the dynamic behaviour of lymph node stromal cells during immune responses. However, once we see deeply, perturbing the system to determine the causalities and functional mechanisms in stromal cell behaviour will be another technical challenge. Image acquisition times and data analysis software limit higher-order analysis of individual cells in whole tissues. Increased access to upcoming novel technology platforms will surpass this obstacle. Stromal immunologists should train in conducting whole-tissue dynamic microscopy and in image analysis of these datasets. Understanding how the behaviour of single cells emerges to produce tissue system functions means that we can begin to explore inter-organ communication at the cellular resolution and intersystem level.

### Tissue remodelling in inflammation

The lymph node tissue structure can expand rapidly in size during adaptive immune responses. Throughout tissue expansion, the stromal architecture is topologically robust and maintains function (Martinez et al., 2019; Novkovic et al., 2016, 2020). CLEC-2 is expressed by mature antigen-presenting DCs and binds to PDPN on FRCs. This interaction causes a loss of actomyosin contractility in FRCs and a relaxation of the FRC network. This allows lymph nodes to accommodate the trapping and clonal expansion of T cells (Acton et al., 2014; Astarita et al., 2015). The network first stretches and elongates before FRCs proliferate. The mechanisms driving FRC proliferation are unknown; however, its timing varies depending on the inflammatory cue, suggesting that signals from the microenvironment (Fletcher et al., 2015) or, as Horsnell et al. describe in a recent preprint, mechanical cues are the drivers of a synchronous organ-wide response (Horsnell et al., 2021 preprint).

Following the resolution of an immune response, lymph nodes return to steady-state size. This occurs after repeated immune challenges without tissue damage and fibrosis. However, the mechanisms controlling the return to the homeostatic state are not well understood (Acton et al., 2014; Astarita et al., 2015). Despite rapid progress in understanding the lymph node stroma and the crosstalk that drives homeostasis, there are still many unexplored topics. Soluble factors might prime lymph node stroma via conduits; however, it is technically challenging to measure this directly (Box 2). Moreover, some infections and pathological states can disrupt lymph node architecture. Changes to FRC phenotype and disruption of the FRC network can lead to loss of tissue function and severe consequences for immune responses. Immune responses are finely balanced, and breakdown of stroma–leukocyte crosstalk can be detrimental by skewing immune responses and enhancing disease severity.

### Viral infections driving disease through lymphoid tissue

Influenza is linked to 650,000 deaths annually, particularly affecting infants and the aged (Iuliano et al., 2018). Ageing has a detrimental effect on both natural immune responses and vaccine-induced immunity against influenza and other viral infections. Newborns rely more on innate responses compared to adults, who depend more on adaptive immune responses (Levy, 2007). In a preprint, Denton et al. showed the importance of stromal cells during influenza virus infection in a mouse model in which T cells from young donors had reduced activation after being transferred into aged mice. This was due to a reduced capacity of aged lymphoid stromal cells to proliferate and retain naïve T cells (Denton et al., 2020 preprint). These findings are in line with previous studies showing that aging negatively impacts immunity (Lefebvre et al., 2012; Masters et al., 2019), but highlight that this immune suppression may also be due to lymphoid tissue stroma dysfunction. Following influenza infection, aged mice have a reduced proliferation of FRCs and diminished T-cell homing due to a decrease in chemokine levels (Masters et al., 2019). Murine studies also show that upon adoptive transfer of young T cells to aged hosts, the aged microenvironment negatively impacts CD4^+^ T-cell homing, antigen detection and development of T follicular helper (Tfh) cells (Lefebvre et al., 2012). Excessive inflammation during influenza virus infection can lead to tissue damage and aberrant tissue repair at the site of infection. Secondary bacterial infections following severe influenza virus and loss of lymph node homeostasis are very common (Herold et al., 2008; Masters et al., 2019; Morris et al., 2017; Pociask et al., 2013), driving morbidity. This supports the notion that an efficient immune response also requires appropriate resolution to prevent secondary infection. Influenza infection models show the importance of immune and stromal cell crosstalk in disease severity (Denton et al., 2014, 2020; Lefebvre et al., 2012; Masters et al., 2019).

Severe acute respiratory syndrome coronavirus 2 (SARS-CoV-2) causes coronavirus disease-19 (COVID-19). Similarly to influenza, COVID-19 severity also increases with age and is influenced by health factors affecting the immune system (Guan et al., 2020). The correlation between disease severity and lymphopenia allows speculations that pathological features are linked to a breakdown in lymph node homeostasis (Guan et al., 2020; Liu et al., 2020; Zhou et al., 2020). The angiotensin-converting enzyme-2 (ACE-2) is a receptor for SARS-CoV-2, while surface lectins such as CD169 (also known as SIGLEC1) enhance ACE-2-dependent infection (Lempp et al., 2021; Li et al., 2003). Human and macaque studies identified SARS-CoV-2 in lymph nodes; however, the field has yet to identify a mechanism by which infection in lymph nodes might drive pathology (Muñoz-Fontela and McElroy, 2017; Polak et al., 2020). Virion-bearing DCs migrate to the lymph nodes, and the virus may use this method to spread systemically (Liu et al., 2016; Muñoz-Fontela and McElroy, 2017). The absence of ACE-2 on lymphoid tissue suggests that direct viral infection is unlikely to drive lymphopenia and that instead, perhaps, it disrupts cell communication within the lymph node (Hamming et al., 2004; Ziegler et al., 2020). Pan and colleagues used immunohistochemistry and immunofluorescence to characterise the spleens and lymph nodes of patients that succumbed to COVID-19 (Pan et al., 2020). CD169^+^ subcapsular sinus macrophages expressed ACE-2 and contained viral nucleoprotein, secreted IL-6 and were highly pro-inflammatory (Pan et al., 2020). A separate post-mortem examination of pulmonary lymph nodes also showed a breakdown in tissue homeostasis, but that was not attributed directly to viral infection (Wang et al., 2021). Instead, T cells were scattered, interfollicular regions widened, and hyperplasia was observed in the vascular and lymphoid sinus endothelial cells (Wang et al., 2021). In lymph nodes, macrophages, LECs and marginal reticular cells interact to support the subcapsular sinus niche. Specifically, LECs provide CSF-1 to maintain the subcapsular sinus macrophages, whereas marginal reticular cells produce RANKL that primes LECs but also homes monocytes to replenish the macrophages lost during inflammation (Camara et al., 2019; Mondor et al., 2019). Infections that cause loss of this macrophage population can disrupt the subcapsular niche, affecting B-cell responses and increasing susceptibility to secondary infections (Gaya et al., 2015). Breakdown of homeostasis and tissue architecture is induced by excessive macrophage inflammation and further enhanced by lymphocyte apoptosis (Pan et al., 2020; Wang et al., 2021).

Disruption of the cellular crosstalk of the tissue niche could have ongoing effects even after an infection has been cleared. The loss of homeostasis may also contribute to enhanced inflammatory responses following SARS-CoV-2 infection, especially in younger patients. A minority of young survivors suffer from autoimmune disorders, Kawasaki-like syndrome (Box 1) and macrophage activation syndrome (Box 1) (Verdoni et al., 2020; Wang et al., 2015). One hypothesis is that the autoimmune and inflammatory syndromes after COVID-19 are triggered by a loss of immune regulation combined with co-infections or environmental factors (Galeotti and Bayry, 2020). A second suggestion is that the SARS-CoV-2 antigens might have homology to self-antigens and thus drive autoimmune responses similar to those observed in Ebola virus (EBOV) syndrome (Galeotti and Bayry, 2020). Overall, these findings show that even if the lymph node stromal cells are not directly targeted by a virus, the changes to their microenvironment can cause a breakdown of homeostasis that leads to loss of tissue architecture and has devastating consequences for the patient.

Connected stromal cell networks can provide a cellular target where viruses can replicate and evade the immune system as they spread through the tissue and beyond. The pathogenicity of haemorrhagic filoviruses, such as EBOV, is induced by systemic viral damage and aberrant immune responses. Lymph node and spleen fibroblasts provide a target for EBOV in macaque models (Steele et al., 2009). Infection of FRCs leads to network damage, conduit dysfunction and loss of lymph node compartmentalisation (Steele et al., 2009; Twenhafel et al., 2013). In the early phases of EBOV infection, viral proteins dampen the recognition by innate immune cells (Muñoz-Fontela and McElroy, 2017; Prescott et al., 2017), as virions travel to lymph nodes within migrating macrophages and DCs, and target FRCs (Baseler et al., 2017; Feldmann et al., 2020; Steele et al., 2009; Twenhafel et al., 2013). EBOV infects the stromal network and spreads systemically to other distal lymph nodes using the lymphatic system (Feldmann et al., 2020). The breakdown of the stromal network has many knock-on effects in pathology, including the apoptosis of lymphocytes in the spleen and lymph nodes through an unidentified mechanism involving intrinsic and extrinsic apoptotic pathways (Baseler et al., 2017; Bradfute et al., 2010; Wauquier et al., 2010). Upon recovery, some EBOV patients show elevated levels of CD8^+^ T cells even a month after the virus was cleared from the plasma (McElroy et al., 2015). These T cells can persist for long periods, suggesting that a breakdown in peripheral tolerance (Box 1) causes tissue damage reminiscent of that observed in graft versus host disease (GVHD; Box 1) (McElroy et al., 2015). Lymphoid stroma plays essential roles in maintaining peripheral tolerance (Fletcher et al., 2010), perhaps explaining how a breakdown in this tissue's homeostasis can lead to ongoing immune dysfunction.

Overall, lymph nodes are key to broadening our understanding of viral infections but also a potential target for modulating immune responses. How viral presence alters the lymph node niches requires further investigation. Understanding how lymphoid tissue architecture responds to infection will inform the development of efficient vaccines and treatments, the improved protection of vulnerable populations and the reduction of aberrant immune responses.

### Loss of lymphoid tissue homeostasis leads to tissue fibrosis

Haematopoietic cell transplantation is a life-saving procedure for millions of patients with haematopoietic disorders. However, it can also lead to devastating aberrant immune responses and tissue fibrosis (Norkin and Wingard, 2017). Patients undergoing such transplantations risk developing acute or chronic GVHD and only half respond to first-line steroid treatment (Khoury et al., 2017). Even successful transplantation can compromise ongoing immune function (Dertschnig et al., 2020). The inflammatory symptoms in GVHD are reminiscent of many multi-organ autoimmune disorders (Zeiser and Blazar, 2017). Allogenic activation of T cells triggers the release of danger-associated molecular patterns by damaged tissue. Breakdown of tissue barrier function in the intestinal epithelium permits entry of pathogen-associated molecular patterns derived from the intestinal bacteria, viruses or fungi (Zelenay and Sousa, 2013). Upon tissue damage in the intestine, bacterial components derived from the gut lumen can enhance acute GVHD (Perkey and Maillard, 2018), and bacterial genome sequencing has shown changes in the microbiome during GVHD progression and treatment (Jenq et al., 2012; Shono et al., 2016). The activation of immune cells driving tissue damage, combined with the presence of danger- and pathogen-associated molecular patterns during the early phases of acute GVHD, maintains a feedback loop that drives irreversible loss of lymphoid tissue homeostasis, causing severe immunodeficiency (Perkey and Maillard, 2018).

**Fig. 2.**
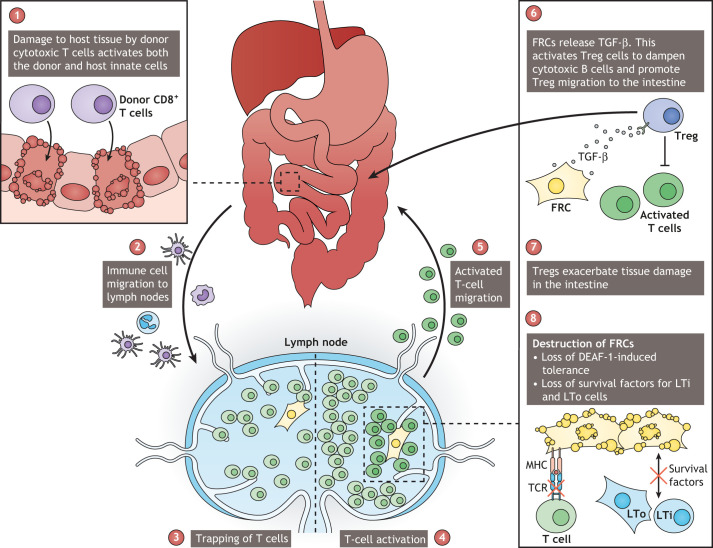
**Breakdown of homeostasis in organ tissue and lymph nodes drives graft versus host disease (GVHD).** (1) Upon allogenic transplantation, donor cells, such as cytotoxic T cells, target host tissue in organs such as the intestine and initiate tissue damage. (2) Innate cells of the host, including neutrophils, monocyte-derived DCs, and donor CD11b^+^ and CD103^+^ DCs migrate to the lymph node. (3) DC recruitment increases the trapping of T cells in the lymph node. (4,5) T cells become activated against cognate host antigens and migrate to the intestine, where they drive damage and remodelling. (6) FRCs in the lymph node secrete TGF-β to activate Tregs and suppress the aberrant activation of T cells. (7) Tregs can migrate to the intestine, modulate the microenvironment, and further exacerbate tissue damage and remodelling. (8) Loss of peripheral tolerance is driven by the aberrant activation of T cells, which target the FRC network. LTi, lymphoid tissue inducer; LTo, lymphoid tissue organiser.

As novel animal models of GVHD are developed, the importance of lymph nodes in its progression is evident. Neutrophils, monocyte-derived cells and donor CD11b^+^ (also known as ITGAM^+^) and CD103^+^ (also known as ITGAE^+^) DCs migrate to the mesenteric lymph nodes, where naïve (CD62L^+^CCR7^+^; also known as SELL^+^CCR7^+^) T cells are activated (Fig. 2) (Koyama et al., 2015). In an attempt to repair the cytotoxic damage, FRCs provide signals, such as TGF-β, that induce regulatory T cells (Tregs) to re-establish lymph node homeostasis (Fig. 2) (Zeiser and Blazar, 2017). Damage to lymphoid tissue also induces the secretion of the alarmins (Box 1) adenosine and IL-33 (Matzinger, 2002; Zeiser and Blazar, 2017). Adenosine activates caspase pathways leading to the release of the bioactive form of IL-1β during acute GVHD (Deaglio et al., 2007; Tsukamoto et al., 2012; Wilhelm et al., 2010), whereas IL-33 and its receptor ST2 (also known as IL1RL1) have been promising targets for multiple autoimmune disorders (Reichenbach et al., 2015; Zeiser and Blazar, 2017; Zhang et al., 2015). Indeed, administration of IL-33 before tissue damage reduces inflammation via binding to the ST2 receptor of Tregs, while administration of IL-33 during the progression of GVHD induces cytotoxic T-cell activation (Reichenbach et al., 2015). Notch signalling (Box 1) is involved in tumours and genetic disorders and is another promising therapeutic target in GVHD (Guruharsha et al., 2012; Sandy et al., 2013; Wood et al., 2015). Both selective inactivation of *Dll1* and *Dll4*, driven by *Ccl19*-Cre, and neutralising antibody experiments in mouse models suggest that, as early as 48 h after hematopoietic cell transplantation, CCL19-expressing FRCs can prime T-cell alloimmunity through DLL-notch ligands (Chung et al., 2017).

Studying the stromal microenvironment has helped appreciate the role that the lymphoid tissue niche plays in the progression of GVHD. As in lymph node development, the interaction between ILC3 and LTo cells might be involved in restoring the FRC network following inflammation or damage (Cupedo and Mebius, 2005; Fletcher et al., 2015). ILC3 cells, like LTi cells, secrete IL-22 to resolve tissue injury in the thymus and intestine, and are a main source of this cytokine during acute GVHD (Dudakov et al., 2012; Hanash et al., 2012). An early influx of LTi and LTo-like cells was observed during acute GVHD; however, neither cell population persists as the disease progresses (Dertschnig et al., 2020). The collapse in lymphoid tissue homeostasis driven by the loss of LTi/LTo function is a potential factor driving the loss of the FRC network through GVHD progression (Fig. 2) (Dertschnig et al., 2020; Simic et al., 2020).

Lymph nodes can induce peripheral T-cell tolerance via the AIRE-like protein DEAF-1 and the expression of tissue-restricted self-antigens (Cohen et al., 2010; Dertschnig et al., 2020; Yip et al., 2009). In the iFABPtOVA mouse model (Box 1), self-antigens are presented by epithelial cells (Fletcher et al., 2010; Vezys et al., 2000). Induction of acute GVHD in iFABPtOVA mice diminished FRC network integrity in a pattern similar to the FRC network depletion seen in OVA.CCL19.DTR.CRE^+/−^ mice (Box 1) in the absence of inflammation (Dertschnig et al., 2020). This underscores the importance of FRCs in maintaining peripheral tolerance and indicates that disruption to the FRC network drives GVHD. These findings also explain the transition from acute to chronic GVHD. The destruction of the FRC network by autoreactive T cells drives the loss of lymph node tissue architecture, which is instrumental for organ functionality (Fig. 2). Chronic GVHD is defined by fibrosis, failure to repair the tissue and allogenic activation of CD8^+^ T cells, which are caused by the destruction of the FRC network and HEVs (Suenaga et al., 2014; Zeiser and Blazar, 2017). Damage to the lymph node stroma causes aberrant activity of effector T cells against peripheral tissues and depletion of humoral immune responses. As a result, patients with chronic GVHD respond poorly to vaccination and have increased risk of recurrent infections. Lymph nodes maintain a response-ready state throughout our lifespan. Understanding how homeostasis collapses during chronic pathologies provides insights for reversing this process.

### Lymph node priming by cancer cells to facilitate metastasis

The metastasis of cancer cells beyond the primary site correlates with poor prognosis. The first site of metastasis is often to tumour-draining lymph nodes or to sentinel lymph nodes (SLNs) (Carlo et al., 2005; Zhang et al., 1997). Primary tumours can promote lymph node metastasis by altering FRC function and skewing it towards an immunosuppressive microenvironment (Cochran et al., 2006; Munn and Mellor, 2006). In murine melanoma, remodelling of the SLN occurred within 11 days after tumour cell injection, and the associated transcriptional reprogramming of FRCs caused a loss in tissue homeostasis (Riedel et al., 2016). Tumour-draining lymph nodes contain FRCs primed towards an activated, pro-fibrotic ‘cancer-associated fibroblast-like’ (CAF-like) state (Riedel et al., 2016). Four CAF subsets (CAF-S1 to CAF-S4) with distinct phenotypes have been identified in breast cancer (Costa et al., 2018). Two of these, CAF-S1 and CAF-S2, are myofibroblast subsets and drive immunosuppression. Specifically, CAF-S1 attract and retain CD4^+^CD25^+^ T cells, whereas both CAF-S1 and CAF-S4 drive the survival of Tregs and dampen effector T-cell activation (Costa et al., 2018). In murine melanoma, CAF-S1 and CAF-S4 were enriched in the SLN compared to the primary tumour. Additionally, the presence of CAF-S1 correlated with epithelial-to-mesenchymal transition and cancer cell invasion, whereas CAF-S4 correlated with ECM remodelling and cancer cell invasion, promoting distant metastasis to other organs (Pelon et al., 2020).

To promote spread to the SLN, the primary tumour induces lymphangiogenesis via expression of VEGF-C and other growth factors (Karaman and Detmar, 2014). Lymphangiogenesis is detectable in the SLN before tumour cells arrive in the tissue (Dadras et al., 2005; Van den Eynden et al., 2006; Hirakawa et al., 2010; Kerjaschki et al., 2011), which contributes to the formation of the pre-metastatic niche. The lymphatic endothelium also provides a protective environment for cancer stem cells and dormant tumour cells (Karaman and Detmar, 2014; Meier et al., 2002).

Tumour metastasis to the SLN is facilitated by chemoattractants expressed by LECs (Karaman and Detmar, 2014). The enhanced lymphatic flow resulting from tumour-induced lymphangiogenesis upregulates CCL21 expression on LECs (Miteva et al., 2010). CCL21 is the ligand for CCR7, which is expressed by several human cancers (Karaman and Detmar, 2014) and correlates with increased lymph node metastasis of breast cancer (Müller et al., 2001). Furthermore, the interaction between CXCL12 on LECs and CXCR4 on invading tumour cells may also promote metastasis to lymph nodes (Hirakawa et al., 2010). The chemokine CCL1, expressed on lymphatic sinuses, is involved during the entry of CCR8-expressing cancer cells into the lymph node, as blocking CCR8 resulted in the arrest of melanoma cells at the lymphatic–subcapsular sinus junctions (Das et al., 2013), showing that cancer cell entry into the lymph node is an actively regulated process. Within the subcapsular sinus, macrophages can capture and present tumour antigens to CD8^+^ T cells (Asano et al., 2018; Singh and Choi, 2019). However, tumour cells may also use cell surface glycans to enter the lymph node by utilising subcapsular sinus macrophages. For example, when hypersialylated melanoma cells reach the lymph node capsule, their interaction with resident macrophages drives cell cycle progression and promotes lymph node metastasis (Singh and Choi, 2019). These findings suggest an organ-specific trait of melanoma to achieve lymph node metastasis.

Cancer cell-mediated remodelling of the SLN also affects its compartmentalisation and the equilibrium of immune cells. Reprogramming of FRCs into CAFs induces a loss of SLN organisation, triggering larger B-cell follicles, a smaller T-cell area and an immunosuppressive microenvironment (Riedel et al., 2016). In breast cancer patients, tumour cell invasion is associated with increase in Tregs in both the primary tumour and the SLN (Núñez et al., 2020). These findings provide strong evidence that the tumour alters the SLN into an immunosuppressive environment. However, it is not completely clear how the primary tumour remodels the SLN before the cancer cells arrive in the lymph node. It is suggested that soluble factors secreted by the primary tumour or tumour-derived exosomes drain to the SLN via the lymphatics to induce a loss of lymphoid tissue homeostasis (Karaman and Detmar, 2014; Riedel et al., 2016). Future studies will need to focus on mechanistically linking the events described here and unravel which mechanisms the primary tumour uses to alter their microenvironment to gain access to lymph nodes and further metastasise.

CAFs can modulate cancer progression through direct or indirect mechanisms. These include ECM production and remodelling, antigen presentation and the secretion of mediators. Overall, these modulate immune cell migration and activation, and support tolerogenic phenotypes (Kraman et al., 2010; Monteran and Erez, 2019; Sahai et al., 2020). CAFs secrete growth factors (HGF, IGF1), tumour-promoting ligands (TFG-β, IL-6 and LIF) and immunosuppressive ligands (CXCL12 and CCL2) (Biffi and Tuveson, 2021; Kraman et al., 2010; Sahai et al., 2020). Mounting evidence suggests that CAFs arise from quiescent fibroblasts populating resident or distant tissues, which become primed and irreversibly activated in response to cues from the tumour microenvironment (Biffi and Tuveson, 2021; LeBleu and Kalluri, 2018). DNA damage after radiotherapy, oxidative stress or changes to physical properties can drive CAF activation (Albrengues et al., 2015; Anderberg and Pietras, 2009; Bai et al., 2015; Calvo et al., 2013; Sahai et al., 2020; Sanz-Moreno et al., 2011). Cancer cells can dictate CAF activity and their organisation within the tumour. High levels of CCL21 expression, which is often upregulated in invasive cancer cells (Shields et al., 2010), induce the formation of stromal-like structures in mouse melanoma tumours that resemble those formed by FRCs in the lymph node. These structures promote a strong tolerogenic response capable of preventing the rejection of non-syngeneic allografts (Shields et al., 2010). However, although the relevance of CAF activation in exerting tolerogenic and anti-cancer functions has been described in detail, it is still unclear how and when CAFs become activated and, perhaps most importantly, what determines their conversion into tumour-promoting or alternatively into tumour-protecting CAFs and how they affect anti-tumour immunity.

### Tertiary lymphoid structures

**Fig. 3.**
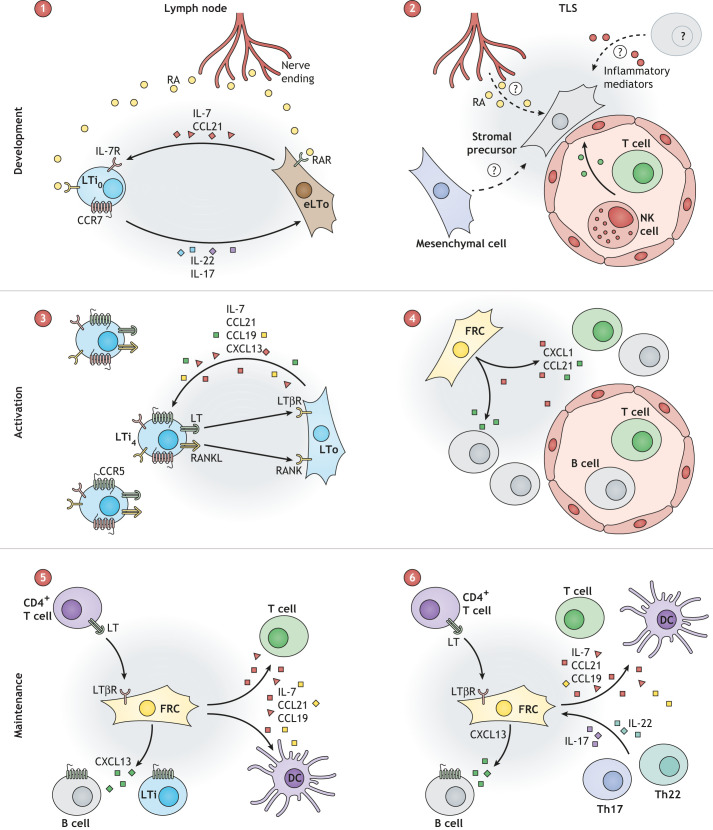
**FRCs drive the formation and maintenance of the niche in lymph nodes and in tertiary lymphoid structures.** (1) Lymph node anlagen formation is driven by the retinoic acid (RA) released by nerves binding to the RA receptor (RAR) on the embryonic lymphoid tissue organiser (eLTo) cells. eLTo cells secrete CCL21 and IL-7, leading to the recruitment and survival of LTi precursors (LTi_0_), which express IL-7 receptor (IL-7R), CCR7 and RAR. LTi_0_ cells secrete IL-22 and IL-17, inducing eLTo differentiation. (2) Tertiary lymphoid structure (TLS) development is not fully understood, but occurs through a phenotypic change of stromal precursors or pericytes proximal to blood vessels. Multiple factors are involved in TLS formation, including IFN-γ [from activated T and natural killer (NK) cells] or inflammatory mediators such as IFN-α or IL-17, while a contribution of RA from neurons cannot be excluded. (3) LTo cells secrete CXCL13, CCL21, CCL19 and IL-7 to recruit LTi_4_ cells. RANKL–RANK and lymphotoxin–LTβR binding between LTi_4_ and LTo cells causes the activation of LTo cells. (4) TLS niche formation is driven by podoplanin^+^ (PDPN^+^) FRCs producing CXCL13 and CCL21 to recruit T and B cells. (5,6) FRCs are central for niche maintenance in lymph nodes and TLSs by interacting with leukocytes. How TLS stroma is replenished is unknown; however, the presence of mesenchymal precursors or LTi/ILC3 cells cannot be excluded. Th, T helper.

In chronic inflammatory conditions, TLSs arise spontaneously by creating a niche like that of secondary lymphoid organs. How TLSs arise and how they are maintained is not fully understood; however, many aspects recapitulate lymph node development and replenishment (Fig. 3). TLS formation is initiated adjacent to blood vessels and is defined by the upregulation of PDPN on stromal cells (Engelhard et al., 2018; Pikor et al., 2015a; Tang et al., 2017). PDPN-expressing fibroblasts in TLSs secrete CXCL13 and CCL21, which drive the recruitment of B and T cells to the proximal tissue. The cells that drive this conversion are unknown and may vary in different pathologies. It is suggested that changes to the perivascular pericytes or to mesenchymal precursors in proximal adipose tissue might be the source of these PDPN^+^ stromal cells (Engelhard et al., 2018).

During influenza virus infection, the presence of type I IFNs and IL-17 has been linked to the presence of TLSs (Denton et al., 2019; Rangel-Moreno et al., 2011). In murine melanoma, the development of tumour-associated TLSs might be driven by PDPN^+^ CAFs that are similar to LTo cells, sensitive to TNFR signalling and secrete CXCL13 (Rodriguez et al., 2021). The formation of TLSs appears to crucially depend on CD4^+^ T-helper (Th) cells (Box 1), whereas the maintenance of PDPN^+^ fibroblasts was highly dependent on lymphotoxin in numerous disease models (Barone et al., 2016; Kang et al., 2002; Pipi et al., 2018).

The interplay between two Th cell subtypes, Th17 and Th21, is also important for shaping the TLS elements. Th17 cells share many of the markers expressed by LTi cells and might be important for stimulating fibroblasts to act as LTo cells (Peters et al., 2011). As TLS development is studied in more detail, researchers should perhaps consider a more active role of the LTi-like ILC3 cells. Interestingly, IL-17 and IL-22 are crucial for the survival of FRCs in TLSs (Barone et al., 2016; Grogan and Ouyang, 2012; Pikor et al., 2015b). Furthermore, TLS FRCs also secrete IL-6 and IL-23, which support Th17 maintenance in an interaction that is highly reminiscent of that between LTi and LTo cells during lymph node development (Mitsdoerffer and Peters, 2016). The lymph nodes provide the ideal model to study how the stromal cells in TLSs are replenished (Box 2). LTo-like FAP^+^ cells have been associated with TLS formation (Denton et al., 2019). However, as in lymph nodes, other stromal precursors derived from proximal adipose tissue or from marginal reticular cells cannot be excluded. In all, TLS presence in a particular disease is associated with a highly variable prognosis that is highly dependent on the inflammatory profile of the pathology. Many parallels can be drawn between the development and homeostasis of lymph nodes and TLSs, and improved understanding of these similarities can provide a promising avenue for therapeutic intervention during chronic diseases.

## Conclusions

Changes to the lymphoid structures and function are driven by infection, cancer and chronic inflammatory disorders. By summarising multiple models of pathologies, this Review highlights the importance of stromal cells in maintaining the lymph node niche and driving immune responses. Homeostasis in the lymph node is generally robust and maintained through the interaction between stromal cells and leukocytes. FRC plasticity is extremely important for mounting efficient immune responses, but can be a detrimental factor in many diseases and chronic disorders if changes in cell state are not reversed. Tumours can alter the microenvironment of lymph nodes to spread systemically. Furthermore, they can alter the phenotype of peripheral fibroblasts to CAFs, which helps support the cancer niche. Viruses have evolved mechanisms to utilise the FRC network to evade the immune system. Severe infection caused by EBOV can change the phenotype of FRCs and induce a loss of efficient adaptive immune responses. Although preliminary, similar patterns are observed in the morbidity induced by SARS-CoV-2 infection.

The homeostasis of lymph node stroma appears to determine the progression of diseases and disorders. Although lymph nodes are pivotal for protection against pathogens, changes in FRC functionality reduce the capability of mounting efficient immune responses. Several factors can damage FRC function, but the outcome is devastating for the host. Loss of FRC function can occur by direct damage, as in viral infections, or through a breakdown in cellular interactions/signals. Loss of FRCs during GVHD is induced by a breakdown of peripheral tolerance, which might also affect tissue repair by skewing the survival factors for LTo and LTi cells. Although great progress has been made in studying lymph node expansion, our understanding of how the tissue returns to the steady state and how the stromal network remains intact following immune responses remains very limited. The identification of additional stromal cell markers will lead to the development of improved transgenic models for studying the role of the lymph node stroma and for understanding the mechanisms involved in lymph node expansion and resolution.

The function of stromal cells is driven by the signals received within the niche. Lymphotoxin from LTi or T cells appears to be an important factor for maintaining the FRC network. In turn, stromal cells maintain the T-cell niche. Interestingly, when receiving the correct signals, FRCs can maintain lymphocytes even outside secondary lymphoid organs. Understanding how TLSs develop is important as they can influence the outcome of many diseases.

Lymphoid stromal cells are highly adaptive and can change their function during immune challenges. This is especially evident during an immune response when lymphocytes proliferate in the lymph nodes. The dynamic plasticity of lymphoid tissue is also exemplified during resolution of immune responses, as the lymph node tissue can return to a steady state and remain primed for any future challenge. Understanding the mechanisms that drive the homeostasis of stromal cells can have multiple benefits in various infections, chronic disorders or cancer and across all age groups. Targeting and modulating the phenotype of stromal cells could provide an essential therapeutic avenue for these disorders and spearhead the development of more efficient treatments and vaccines.

## References

[DMM049256C1] Acton, S. E., Astarita, J. L., Malhotra, D., Lukacs-Kornek, V., Franz, B., Hess, P. R., Jakus, Z., Kuligowski, M., Fletcher, A. L., Elpek, K. G. et al. (2012). Podoplanin-rich stromal networks induce dendritic cell motility via activation of the C-type lectin receptor CLEC-2. *Immunity* 37, 276-289. 10.1016/j.immuni.2012.05.022 22884313 PMC3556784

[DMM049256C2] Acton, S. E., Farrugia, A. J., Astarita, J. L., Mourão-Sá, D., Jenkins, R. P., Nye, E., Hooper, S., van Blijswijk, J., Rogers, N. C., Snelgrove, K. J. et al. (2014). Dendritic cells control fibroblastic reticular network tension and lymph node expansion. *Nature* 514, 498-502. 10.1038/nature13814 25341788 PMC4235005

[DMM049256C3] Acton, S. E., Onder, L., Novkovic, M., Martinez, V. G. and Ludewig, B. (2021). Communication, construction, and fluid control: lymphoid organ fibroblastic reticular cell and conduit networks. *Trends Immunol.* 42, 782-794. 10.1016/j.it.2021.07.003 34362676

[DMM049256C4] Ager, A. (2017). High endothelial venules and other blood vessels: critical regulators of lymphoid organ development and function. *Front. Immunol.* 8, 45. 10.3389/fimmu.2017.00045 28217126 PMC5289948

[DMM049256C5] Albrengues, J., Bertero, T., Grasset, E., Bonan, S., Maiel, M., Bourget, I., Philippe, C., Serrano, C. H., Benamar, S., Croce, O. et al. (2015). Epigenetic switch drives the conversion of fibroblasts into proinvasive cancer-associated fibroblasts. *Nat. Commun.* 6, 10204-10215. 10.1038/ncomms10204 26667266 PMC4682161

[DMM049256C6] Anderberg, C. and Pietras, K. (2009). On the origin of cancer-associated fibroblasts. *Cell Cycle* 8, 1461-1465. 10.4161/cc.8.10.8557 19395855

[DMM049256C7] Asano, T., Ohnishi, K., Shiota, T., Motoshima, T., Sugiyama, Y., Yatsuda, J., Kamba, T., Ishizaka, K. and Komohara, Y. (2018). CD169-positive sinus macrophages in the lymph nodes determine bladder cancer prognosis. *Cancer Sci.* 109, 1723-1730. 10.1111/cas.13565 29520898 PMC5980134

[DMM049256C8] Astarita, J. L., Cremasco, V., Fu, J., Darnell, M. C., Peck, J. R., Nieves-Bonilla, J. M., Song, K., Kondo, Y., Woodruff, M. C., Gogineni, A. et al. (2015). The CLEC-2–podoplanin axis controls the contractility of fibroblastic reticular cells and lymph node microarchitecture. *Nat. Immunol.* 16, 75-84. 10.1038/ni.3035 25347465 PMC4270928

[DMM049256C9] Bai, L., Wang, F., Zhang, D. S., Li, C., Jin, Y., Wang, D. S., Chen, D. L., Qiu, M. Z., Luo, H. Y., Wang, Z. Q. et al. (2015). A plasma cytokine and angiogenic factor (CAF) analysis for selection of bevacizumab therapy in patients with metastatic colorectal cancer. *Sci. Rep.* 5, 1-12. 10.1038/srep17717 PMC4664961 26620439

[DMM049256C10] Bajénoff, M., Egen, J. G., Koo, L. Y., Laugier, J. P., Brau, F., Glaichenhaus, N. and Germain, R. N. (2006). Stromal cell networks regulate lymphocyte entry, migration, and territoriality in lymph nodes. *Immunity* 25, 989-1001. 10.1016/j.immuni.2006.10.011 17112751 PMC2692293

[DMM049256C11] Barbazan, J. and Vignjevic, D. M. (2019). Cancer associated fibroblasts: is the force the path to the dark side? *Curr. Opin. Cell Biol.* 56, 71-79. 10.1016/j.ceb.2018.09.002 30308331

[DMM049256C12] Barone, F., Gardner, D. H., Nayar, S., Steinthal, N., Buckley, C. D. and Luther, S. A. (2016). Stromal fibroblasts in tertiary lymphoid structures: A novel target in chronic inflammation. *Front. Immunol.* 7, 477. 10.3389/fimmu.2016.00477 27877173 PMC5100680

[DMM049256C13] Baseler, L., Chertow, D. S., Johnson, K. M., Feldmann, H. and Morens, D. M. (2017). The pathogenesis of Ebola virus disease. *Annu. Rev. Pathol.* 12, 387-418. 10.1146/annurev-pathol-052016-100506 27959626

[DMM049256C14] Bénézech, C., White, A., Mader, E., Serre, K., Parnell, S., Pfeffer, K., Ware, C. F., Anderson, G. and Caamaño, J. H. (2010). Ontogeny of stromal organizer cells during lymph node development. *J. Immunol.* 184, 4521-4530. 10.4049/jimmunol.0903113 20237296 PMC2862734

[DMM049256C15] Bénézech, C., Mader, E., Desanti, G., Khan, M., Nakamura, K., White, A., Ware, C. F., Anderson, G. and Caamaño, J. H. (2012). Lymphotoxin-β receptor signaling through NF-κB2-RelB pathway reprograms adipocyte precursors as lymph node stromal cells. *Immunity* 37, 721-734. 10.1016/j.immuni.2012.06.010 22940098 PMC3809035

[DMM049256C16] Biffi, G. and Tuveson, D. A. (2021). Diversity and biology of cancer-associated fibroblasts. *Physiol. Rev.* 101, 147-176. 10.1152/physrev.00048.2019 32466724 PMC7864232

[DMM049256C17] Bovay, E., Sabine, A., Prat-Luri, B., Kim, S., Son, K., Willrodt, A.-H., Olsson, C., Halin, C., Kiefer, F., Betsholtz, C. et al. (2018). Multiple roles of lymphatic vessels in peripheral lymph node development. *J. Exp. Med.* 215, 2760-2777. 10.1084/jem.20180217 30355615 PMC6219737

[DMM049256C18] Bradfute, S. B., Swanson, P. E., Smith, M. A., Watanabe, E., McDunn, J. E., Hotchkiss, R. S. and Bavari, S. (2010). Mechanisms and consequences of ebolavirus-induced lymphocyte apoptosis. *J. Immunol.* 184, 327-335. 10.4049/jimmunol.0901231 20028660

[DMM049256C19] Brendolan, A. and Caamaño, J. H. (2012). Mesenchymal cell differentiation during lymph node organogenesis. *Front. Immunol.* 3, 381. 10.3389/fimmu.2012.00381 23248630 PMC3522075

[DMM049256C168] Brown, F. D., Sen, D. R., LaFleur, M. W., Godec, J., Lukacs-Kornek, V., Schildberg, F. A., Kim, H.-J., Yates, K. B., Ricoult, S. J. H., Bi, K. et al. (2019). Fibroblastic reticular cells enhance T cell metabolism and survival via epigenetic remodeling. *Nat. Immunol*. 20, 1668-1680. 10.1038/s41590-019-0515-x 31636464 PMC7464610

[DMM049256C20] Brulois, K., Rajaraman, A., Szade, A., Nordling, S., Bogoslowski, A., Dermadi, D., Rahman, M., Kiefel, H., O'Hara, E., Koning, J. J. et al. (2020). A molecular map of murine lymph node blood vascular endothelium at single cell resolution. *Nat. Commun.* 11, 1-15. 10.1038/s41467-020-17291-5 32732867 PMC7393069

[DMM049256C21] Buechler, M. B., Pradhan, R. N., Krishnamurty, A. T., Cox, C., Calviello, A. K., Wang, A. W., Yang, Y. A., Tam, L., Caothien, R., Roose-Girma, M. et al. (2021). Cross-tissue organization of the fibroblast lineage. *Nature* 593, 575-579. 10.1038/s41586-021-03549-5 33981032

[DMM049256C22] Calvo, F., Ege, N., Grande-Garcia, A., Hooper, S., Jenkins, R. P., Chaudhry, S. I., Harrington, K., Williamson, P., Moeendarbary, E., Charras, G. et al. (2013). Mechanotransduction and YAP-dependent matrix remodelling is required for the generation and maintenance of cancer-associated fibroblasts. *Nat. Cell Biol.* 15, 637-646. 10.1038/ncb2756 23708000 PMC3836234

[DMM049256C23] Camara, A., Cordeiro, O. G., Alloush, F., Sponsel, J., Chypre, M., Onder, L., Asano, K., Tanaka, M., Yagita, H., Ludewig, B. et al. (2019). Lymph node mesenchymal and endothelial stromal cells cooperate via the RANK-RANKL cytokine axis to shape the sinusoidal macrophage niche. *Immunity* 50, 1467-1481.e6. 10.1016/j.immuni.2019.05.008 31201093

[DMM049256C24] Carlo, J. T., Grant, M. D., Knox, S. M., Jones, R. C., Hamilton, C. S., Livingston, S. A. and Kuhn, J. A. (2005). Survival analysis following sentinel lymph node biopsy: a validation trial demonstrating its accuracy in staging early breast cancer. *Proc. (Bayl. Univ. Med. Cent.)* 18, 103-107. 10.1080/08998280.2005.11928044 16200155 PMC1200707

[DMM049256C25] Chauveau, A., Pirgova, G., Cheng, H.-W., Martin, A. D., Zhou, F. Y., Wideman, S., Rittscher, J., Ludewig, B. and Arnon, T. I. (2020). Visualization of T cell migration in the spleen reveals a network of perivascular pathways that guide entry into T zones. *Immunity* 52, 794-807.e7. 10.1016/j.immuni.2020.03.010 32298648 PMC7237890

[DMM049256C26] Cho, H., Kim, J., Ahn, J. H., Hong, Y.-K., Mäkinen, T., Lim, D.-S. and Koh, G. Y. (2019). YAP and TAZ negatively regulate Prox1 during developmental and pathologic lymphangiogenesis. *Circ. Res.* 124, 225-242. 10.1161/circresaha.118.313707 30582452

[DMM049256C27] Choi, S. Y., Bae, H., Jeong, S.-H., Park, I., Cho, H., Hong, S. P., Lee, D.-H., Lee, C., Park, J.-S., Suh, S. H. et al. (2020). YAP/TAZ direct commitment and maturation of lymph node fibroblastic reticular cells. *Nat. Commun.* 11, 1-15. 10.1038/s41467-020-14293-1 31980640 PMC6981200

[DMM049256C28] Chung, K. and Deisseroth, K. (2013). CLARITY for mapping the nervous system. *Nat. Methods* 10, 508-513. 10.1038/nmeth.2481 23722210

[DMM049256C29] Chung, J., Ebens, C. L., Perkey, E., Radojcic, V., Koch, U., Scarpellino, L., Tong, A., Allen, F., Wood, S., Feng, J. et al. (2017). Fibroblastic niches prime T cell alloimmunity through Delta-like Notch ligands. *J. Clin. Investig.* 127, 1574-1588. 10.1172/jci89535 28319044 PMC5373885

[DMM049256C30] Cochran, A. J., Huang, R.-R., Lee, J., Itakura, E., Leong, S. P. L. and Essner, R. (2006). Tumour-induced immune modulation of sentinel lymph nodes. *Nature* 6, 659-670. 10.1038/nri1919 16932751

[DMM049256C31] Cohen, J. N., Guidi, C. J., Tewalt, E. F., Qiao, H., Rouhani, S. J., Ruddell, A., Farr, A. G., Tung, K. S. and Engelhard, V. H. (2010). Lymph node–resident lymphatic endothelial cells mediate peripheral tolerance via Aire-independent direct antigen presentation. *J. Exp. Med.* 207, 681-688. 10.1084/jem.20092465 20308365 PMC2856027

[DMM049256C32] Cosgrove, J., Novkovic, M., Albrecht, S., Pikor, N. B., Zhou, Z., Onder, L., Mörbe, U., Cupovic, J., Miller, H., Alden, K. et al. (2020). B cell zone reticular cell microenvironments shape CXCL13 gradient formation. *Nat. Commun.* 11, 1-15. 10.1038/s41467-020-17135-2 32699279 PMC7376062

[DMM049256C33] Costa, A., Kieffer, Y., Scholer-Dahirel, A., Pelon, F., Bourachot, B., Cardon, M., Sirven, P., Magagna, I., Fuhrmann, L., Bernard, C. et al. (2018). Fibroblast heterogeneity and immunosuppressive environment in human breast cancer. *Cancer Cell* 33, 463-479.e10. 10.1016/j.ccell.2018.01.011 29455927

[DMM049256C34] Cremasco, V., Woodruff, M. C., Onder, L., Cupovic, J., Nieves-Bonilla, J. M., Schildberg, F. A., Chang, J., Cremasco, F., Harvey, C. J., Wucherpfennig, K. et al. (2014). B cell homeostasis and follicle confines are governed by fibroblastic reticular cells. *Nat. Immunol.* 15, 973-981. 10.1038/ni.2965 25151489 PMC4205585

[DMM049256C35] Cui, L., Chen, S.-Y., Lerbs, T., Lee, J.-W., Domizi, P., Gordon, S., Kim, Y., Nolan, G., Betancur, P. and Wernig, G. (2020). Activation of JUN in fibroblasts promotes pro-fibrotic programme and modulates protective immunity. 11, 2795. 10.1038/s41467-020-16466-4 PMC7270081 32493933

[DMM049256C36] Cupedo, T. and Mebius, R. E. (2005). Cellular interactions in lymph node development. *J. Immunol.* 174, 21-25. 10.4049/jimmunol.174.1.21 15611222

[DMM049256C37] Cupedo, T., Vondenhoff, M. F. R., Heeregrave, E. J., de Weerd, A. E., Jansen, W., Jackson, D. G., Kraal, G. and Mebius, R. E. (2004). Presumptive lymph node organizers are differentially represented in developing mesenteric and peripheral nodes. *J. Immunol.* 173, 2968-2975. 10.4049/jimmunol.173.5.2968 15322155

[DMM049256C38] Da Graça, C. G., van Baarsen, L. G. M. and Mebius, R. E. (2021). Tertiary lymphoid structures: diversity in their development, composition, and role. *J. Immunol.* 206, 273-281. 10.4049/jimmunol.2000873 33397741

[DMM049256C39] Dadras, S. S., Lange-Asschenfeldt, B., Velasco, P., Nguyen, L., Vora, A., Muzikansky, A., Jahnke, K., Hauschild, A., Hirakawa, S., Mihm, M. C. et al. (2005). Tumor lymphangiogenesis predicts melanoma metastasis to sentinel lymph nodes. *Mod. Pathol.*,18, 1232-1242. 10.1038/modpathol.3800410 15803182

[DMM049256C40] Das, S., Sarrou, E., Podgrabinska, S., Cassella, M., Mungamuri, S. K., Feirt, N., Gordon, R., Nagi, C. S., Wang, Y., Entenberg, D. et al. (2013). Tumor cell entry into the lymph node is controlled by CCL1 chemokine expressed by lymph node lymphatic sinuses. *J. Exp. Med.* 210, 1509-1528. 10.1084/jem.20111627 23878309 PMC3727324

[DMM049256C41] Deaglio, S., Dwyer, K. M., Gao, W., Friedman, D., Usheva, A., Erat, A., Chen, J.-F., Enjyoji, K., Linden, J., Oukka, M. et al. (2007). Adenosine generation catalyzed by CD39 and CD73 expressed on regulatory T cells mediates immune suppression. *J. Exp. Med.* 204, 1257-1265. 10.1084/jem.20062512 17502665 PMC2118603

[DMM049256C42] Denton, A. E., Roberts, E. W., Linterman, M. A. and Fearon, D. T. (2014). Fibroblastic reticular cells of the lymph node are required for retention of resting but not activated CD8+ T cells. *Proc. Natl Acad. Sci. USA* 111, 12139-12144. 10.1073/pnas.1412910111 25092322 PMC4143042

[DMM049256C43] Denton, A. E., Carr, E. J., Magiera, L. P., Watts, A. J. B. and Fearon, D. T. (2019). Embryonic FAP^+^ lymphoid tissue organizer cells generate the reticular network of adult lymph nodes. *J. Exp. Med.* 216, 2242-2252. 10.1084/jem.20181705 31324739 PMC6780995

[DMM049256C44] Denton, A. E., Silva-Cayetano, A., Dooley, J., Hill, D. L., Carr, E. J., Robert, P. A., Meyer-Hermann, M., Liston, A. and Linterman, M. A. (2020). Intrinsic defects in lymph node stromal cells underpin poor germinal center responses during aging. *BioRxiv* 2020.05.07.082255. 10.1101/2020.05.07.082255

[DMM049256C45] Dertschnig, S., Evans, P., Sousa, P. S. E., Manzo, T., Ferrer, I. R., Stauss, H. J., Bennett, C. L. and Chakraverty, R. (2020). Graft-versus-host disease reduces lymph node display of tissue-restricted self-antigens and promotes autoimmunity. *J. Clin. Investig.* 130, 1896-1911. 10.1172/jci133102 31917684 PMC7108931

[DMM049256C46] Dudakov, J. A., Hanash, A. M., Jenq, R. R., Young, L. F., Ghosh, A., Singer, N. V., West, M. L., Smith, O. M., Holland, A. M., Tsai, J. J. et al. (2012). Interleukin-22 drives endogenous thymic regeneration in mice. *Science* 336, 91-95. 10.1126/science.1218004 22383805 PMC3616391

[DMM049256C47] Eberl, G., Marmon, S., Sunshine, M.-J., Rennert, P. D., Choi, Y. and Littman, D. R. (2003). An essential function for the nuclear receptor RORγt in the generation of fetal lymphoid tissue inducer cells. *Nat. Immunol.* 5, 64-73. 10.1038/ni1022 14691482

[DMM049256C48] Engelhard, V. H., Rodriguez, A. B., Mauldin, I. S., Woods, A. N., Peske, J. D. and Slingluff, C. L. (2018). Immune cell infiltration and tertiary lymphoid structures as determinants of antitumor immunity. *J. Immunol.* 200, 432-442. 10.4049/jimmunol.1701269 29311385 PMC5777336

[DMM049256C49] Entenberg, D., Pastoriza, J. M., Oktay, M. H., Voiculescu, S., Wang, Y., Sosa, M. S., Aguirre-Ghiso, J. and Condeelis, J. (2017). Time-lapsed, large-volume, high-resolution intravital imaging for tissue-wide analysis of single cell dynamics. *Methods* 128, 65-77. 10.1016/j.ymeth.2017.07.019 28911733 PMC5659295

[DMM049256C50] Feldmann, H., Sprecher, A. and Geisbert, T. W. (2020). Ebola. *N. Engl. J. Med.* 382, 1832-1842. 10.1056/nejmra1901594 32441897

[DMM049256C51] Fletcher, A. L., Acton, S. E. and Knoblich, K. (2015). Lymph node fibroblastic reticular cells in health and disease. *Nature* 15, 1-12. 10.1038/nri3846 PMC5152733 25998961

[DMM049256C52] Fletcher, A. L., Lukacs-Kornek, V., Reynoso, E. D., Pinner, S. E., Bellemare-Pelletier, A., Curry, M. S., Collier, A.-R., Boyd, R. L. and Turley, S. J. (2010). Lymph node fibroblastic reticular cells directly present peripheral tissue antigen under steady-state and inflammatory conditions. *J. Exp. Med.* 207, 689-697. 10.1084/jem.20092642 20308362 PMC2856033

[DMM049256C53] Förster, R., Schubel, A., Breitfeld, D., Kremmer, E., Renner-Müller, I., Wolf, E. and Lipp, M. (1999). CCR7 coordinates the primary immune response by establishing functional microenvironments in secondary lymphoid organs. *Cell* 99, 23-33. 10.1016/s0092-8674(00)80059-8 10520991

[DMM049256C54] Fujimoto, N., He, Y., D'Addio, M., Tacconi, C., Detmar, M. and Dieterich, L. C. (2020). Single-cell mapping reveals new markers and functions of lymphatic endothelial cells in lymph nodes. *PLoS Biol.* 18, e3000704-e24. 10.1371/journal.pbio.3000704 32251437 PMC7162550

[DMM049256C55] Galeotti, C. and Bayry, J. (2020). Autoimmune and inflammatory diseases following COVID-19. *Nat. Rev. Rheumatol.* 395, 413-413. 10.1038/s41584-020-0448-7 PMC7271827 32499548

[DMM049256C56] Gaya, M., Castello, A., Montaner, B., Rogers, N., Sousa, C. R. E., Bruckbauer, A. and Batista, F. D. (2015). Host response. Inflammation-induced disruption of SCS macrophages impairs B cell responses to secondary infection. *Science* 347, 667-672. 10.1126/science.aaa1300 25657250

[DMM049256C57] Gil-Ortega, M., Garidou, L., Barreau, C., Maumus, M., Breasson, L., Tavernier, G., García-Prieto, C. F., Bouloumié, A., Casteilla, L. and Sengenès, C. (2013). Native adipose stromal cells egress from adipose tissue in vivo: evidence during lymph node activation. *Stem Cells* 31, 1309-1320. 10.1002/stem.1375 23533182

[DMM049256C58] Girard, J.-P., Moussion, C. and Förster, R. (2012). HEVs, lymphatics and homeostatic immune cell trafficking in lymph nodes. *Nat. Rev. Immunol.* 12, 762-763. 10.1038/nri3298 23018291

[DMM049256C59] Grogan, J. L. and Ouyang, W. (2012). A role for Th17 cells in the regulation of tertiary lymphoid follicles. *Eur. J. Immunol.* 42, 2255-2262. 10.1002/eji.201242656 22949324

[DMM049256C60] Guan, W., Ni, Z., Hu, Y., Liang, W., Ou, C., He, J., Liu, L., Shan, H., Lei, C., Hui, D. S. C. et al. (2020). Clinical characteristics of coronavirus disease 2019 in China. *N. Engl. J. Med.* 382, 1708-1720. 10.1056/nejmoa2002032 32109013 PMC7092819

[DMM049256C61] Gunn, M. D., Kyuwa, S., Tam, C., Kakiuchi, T., Matsuzawa, A., Williams, L. T. and Nakano, H. (1999). Mice lacking expression of secondary lymphoid organ chemokine have defects in lymphocyte homing and dendritic cell localization. *J. Exp. Med.*,189, 451-460. 10.1084/jem.189.3.451 9927507 PMC2192914

[DMM049256C62] Guruharsha, K. G., Kankel, M. W. and Artavanis-Tsakonas, S. (2012). The Notch signalling system: recent insights into the complexity of a conserved pathway. *Nat. Rev. Genet.* 13, 654-666. 10.1038/nrg3272 22868267 PMC4369923

[DMM049256C63] Hamming, I., Timens, W., Bulthuis, M., Lely, A. T., Navis, G. J. and van Goor, H. (2004). Tissue distribution of ACE2 protein, the functional receptor for SARS coronavirus. A first step in understanding SARS pathogenesis. *J. Pathol.* 203, 631-637. 10.1002/path.1570 15141377 PMC7167720

[DMM049256C64] Hanash, A. M., Dudakov, J. A., Hua, G., O'Connor, M. H., Young, L. F., Singer, N. V., West, M. L., Jenq, R. R., Holland, A. M., Kappel, L. W. et al. (2012). Interleukin-22 protects intestinal stem cells from immune-mediated tissue damage and regulates sensitivity to graft versus host disease. *Immunity* 37, 339-350. 10.1016/j.immuni.2012.05.028 22921121 PMC3477611

[DMM049256C65] Herold, S., Steinmueller, M., von Wulffen, W., Cakarova, L., Pinto, R., Pleschka, S., Mack, M., Kuziel, W. A., Corazza, N., Brunner, T. et al. (2008). Lung epithelial apoptosis in influenza virus pneumonia: the role of macrophage-expressed TNF-related apoptosis-inducing ligand. *J. Exp. Med.* 205, 3065-3077. 10.1084/jem.20080201 19064696 PMC2605231

[DMM049256C66] Herzog, B. H., Fu, J., Wilson, S. J., Hess, P. R., Sen, A., McDaniel, J. M., Pan, Y., Sheng, M., Yago, T., Silasi-Mansat, R. et al. (2013). Podoplanin maintains high endothelial venule integrity by interacting with platelet CLEC-2. *Nature* 502, 105-109. 10.1038/nature12501 23995678 PMC3791160

[DMM049256C67] Hirakawa, S., Detmar, M., Kerjaschki, D., Nagamatsu, S., Matsuo, K., Tanemura, A., Kamata, N., Higashikawa, K., Okazaki, H., Kameda, K. et al. (2010). Nodal lymphangiogenesis and metastasis. *Am. J. Pathol.* 175, 2235-2248. 10.2353/ajpath.2009.090420 PMC2774085 19815713

[DMM049256C68] Horsnell, H. L., Tetley, R. J., Belly, H. D., Makris, S., Millward, L. J., Benjamin, A. C., de Winde, C. M., Paluch, E. K., Mao, Y. and Acton, S. E. (2021). Tissue homeostasis and adaptation to immune challenge resolved by fibroblast network mechanics. *BioRxiv* 2021.05.27.446027. 10.1101/2021.05.27.446027 PMC9355877 35882934

[DMM049256C69] Huang, H.-Y., Rivas-Caicedo, A., Renevey, F., Cannelle, H., Peranzoni, E., Scarpellino, L., Hardie, D. L., Pommier, A., Schaeuble, K., Favre, S. et al. (2018). Identification of a new subset of lymph node stromal cells involved in regulating plasma cell homeostasis. *Proc. Natl. Acad. Sci. U.S.A.* 115, E6826-E6835. 10.1073/pnas.1712628115 29967180 PMC6055158

[DMM049256C70] Iuliano, A. D., Roguski, K. M., Chang, H. H., Muscatello, D. J., Palekar, R., Tempia, S., Cohen, C., Gran, J. M., Schanzer, D., Cowling, B. J. et al. (2018). Estimates of global seasonal influenza-associated respiratory mortality: a modelling study. *Lancet* 391, 1285-1300. 10.1016/s0140-6736(17)33293-2 29248255 PMC5935243

[DMM049256C71] Jalkanen, S. and Salmi, M. (2020). Lymphatic endothelial cells of the lymph node. *Nat. Rev. Immunol.* 20, 566-578. 10.1038/s41577-020-0281-x 32094869

[DMM049256C72] Jenq, R. R., Ubeda, C., Taur, Y., Menezes, C. C., Khanin, R., Dudakov, J. A., Liu, C., West, M. L., Singer, N. V., Equinda, M. J. et al. (2012). Regulation of intestinal inflammation by microbiota following allogeneic bone marrow transplantation. *J. Exp. Med.* 209, 903-911. 10.1084/jem.20112408 22547653 PMC3348096

[DMM049256C73] Jones, E., Gallimore, A. and Ager, A. (2018). Defining high endothelial venules and tertiary lymphoid structures in cancer. *Methods Mol. Biol.* 1845, 99-118. 10.1007/978-1-4939-8709-2_7 30141010

[DMM049256C74] Kaldjian, E. P., Gretz, J. E., Anderson, A. O., Shi, Y. and Shaw, S. (2001). Spatial and molecular organization of lymph node T cell cortex: a labyrinthine cavity bounded by an epithelium-like monolayer of fibroblastic reticular cells anchored to basement membrane-like extracellular matrix. *Int. Immunol.* 13, 1243-1253. 10.1093/intimm/13.10.1243 11581169

[DMM049256C75] Kang, Y. M., Zhang, X., Wagner, U. G., Yang, H., Beckenbaugh, R. D., Kurtin, P. J., Goronzy, J. J. and Weyand, C. M. (2002). CD8 T cells are required for the formation of ectopic germinal centers in rheumatoid synovitis. *J. Exp. Med.* 195, 1325-1336. 10.1084/jem.20011565 12021312 PMC2193749

[DMM049256C76] Kapoor, V. N., Müller, S., Keerthivasan, S., Brown, M., Chalouni, C., Storm, E. E., Castiglioni, A., Lane, R., Nitschke, M., Dominguez, C. X. et al. (2021). Gremlin 1^+^ fibroblastic niche maintains dendritic cell homeostasis in lymphoid tissues. *Nat. Immunol.* 22, 571-585. 10.1038/s41590-021-00920-6 33903764

[DMM049256C77] Karaman, S. and Detmar, M. (2014). Mechanisms of lymphatic metastasis. *J. Clin. Investig.* 124, 922-928. 10.1172/jci71606 24590277 PMC3938272

[DMM049256C78] Katakai, T., Suto, H., Sugai, M., Gonda, H., Togawa, A., Suematsu, S., Ebisuno, Y., Katagiri, K., Kinashi, T. and Shimizu, A. (2008). Organizer-like reticular stromal cell layer common to adult secondary lymphoid organs. *J. Immunol.* 181, 6189-6200. 10.4049/jimmunol.181.9.6189 18941209

[DMM049256C79] Kerjaschki, D., Bago-Horvath, Z., Rudas, M., Sexl, V., Schneckenleithner, C., Wolbank, S., Bartel, G., Krieger, S., Kalt, R., Hantusch, B. et al. (2011). Lipoxygenase mediates invasion of intrametastatic lymphatic vessels and propagates lymph node metastasis of human mammary carcinoma xenografts in mouse. *J. Clin. Investig.* 121, 2000-2012. 10.1172/jci44751 21540548 PMC3083794

[DMM049256C80] Khoury, H. J., Wang, T., Hemmer, M. T., Couriel, D., Alousi, A., Cutler, C., Aljurf, M., Antin, J. H., Ayas, M., Battiwalla, M. et al. (2017). Improved survival after acute graft-versus-host disease diagnosis in the modern era. *Haematologica* 102, 958-966. 10.3324/haematol.2016.156356 28302712 PMC5477615

[DMM049256C81] Koning, J. J., Konijn, T., Lakeman, K. A., O'Toole, T., Kenswil, K. J. G., Raaijmakers, M. H. G. P., Michurina, T. V., Enikolopov, G. and Mebius, R. E. (2016). Nestin-expressing precursors give rise to both endothelial as well as nonendothelial lymph node stromal cells. *J. Immunol.* 197, 2686-2694. 10.4049/jimmunol.1501162 27574301 PMC5459591

[DMM049256C82] Koyama, M., Cheong, M., Markey, K. A., Gartlan, K. H., Kuns, R. D., Locke, K. R., Lineburg, K. E., Teal, B. E., Mouttie, L. L.-E., Bunting, M. D. et al. (2015). Donor colonic CD103^+^ dendritic cells determine the severity of acute graft-versus-host disease. *J. Exp. Med.* 212, 1303-1321. 10.1084/jem.20150329 26169940 PMC4516799

[DMM049256C83] Kraman, M., Bambrough, P. J., Arnold, J. N., Roberts, E. W., Magiera, L., Jones, J. O., Gopinathan, A., Tuveson, D. A. and Fearon, D. T. (2010). Suppression of antitumor immunity by stromal cells expressing fibroblast activation protein–α. *Science* 330, 827-830. 10.1126/science.1195300 21051638

[DMM049256C84] Krishnamurty, A. T. and Turley, S. J. (2020). Lymph node stromal cells: cartographers of the immune system. *Nat. Immunol.* 21, 1-12. 10.1038/s41590-020-0635-3 32205888

[DMM049256C85] LeBleu, V. S. and Kalluri, R. (2018). A peek into cancer-associated fibroblasts: origins, functions and translational impact. *Dis. Model. Mech.* 11, dmm029447. 10.1242/dmm.029447 29686035 PMC5963854

[DMM049256C86] Lee, J.-W., Epardaud, M., Sun, J., Becker, J. E., Cheng, A. C., Yonekura, A., Heath, J. K. and Turley, S. J. (2007). Peripheral antigen display by lymph node stroma promotes T cell tolerance to intestinal self. *Nat. Immunol.* 8, 181-190. 10.1038/ni1427 17195844

[DMM049256C87] Lefebvre, J. S., Maue, A. C., Eaton, S. M., Lanthier, P. A., Tighe, M. and Haynes, L. (2012). The aged microenvironment contributes to the age–related functional defects of CD4 T cells in mice. *Aging Cell* 11, 732-740. 10.1111/j.1474-9726.2012.00836.x 22607653 PMC3444657

[DMM049256C88] Lempp, F. A., Soriaga, L. B., Montiel-Ruiz, M., Benigni, F., Noack, J., Park, Y.-J., Bianchi, S., Walls, A. C., Bowen, J. E., Zhou, J. et al. (2021). Lectins enhance SARS-CoV-2 infection and influence neutralizing antibodies. *Nature* 598, 342-347. 10.1038/s41586-021-03925-1 34464958

[DMM049256C89] Levy, O. (2007). Innate immunity of the newborn: basic mechanisms and clinical correlates. *Nat. Rev. Immunol.* 7, 379-390. 10.1038/nri2075 17457344

[DMM049256C90] Li, W., Moore, M. J., Vasilieva, N., Sui, J., Wong, S. K., Berne, M. A., Somasundaran, M., Sullivan, J. L., Luzuriaga, K., Greenough, T. C. et al. (2003). Angiotensin-converting enzyme 2 is a functional receptor for the SARS coronavirus. *Nature* 426, 450-454. 10.1038/nature02145 14647384 PMC7095016

[DMM049256C91] Link, A., Vogt, T. K., Favre, S., Britschgi, M. R., Acha-Orbea, H., Hinz, B., Cyster, J. G. and Luther, S. A. (2007). Fibroblastic reticular cells in lymph nodes regulate the homeostasis of naive T cells. *Nat. Immunol.* 8, 1255-1265. 10.1038/ni1513 17893676

[DMM049256C92] Liu, L., Wei, Q., Nishiura, K., Peng, J., Wang, H., Midkiff, C., Alvarez, X., Qin, C., Lackner, A. and Chen, Z. (2016). Spatiotemporal interplay of severe acute respiratory syndrome coronavirus and respiratory mucosal cells drives viral dissemination in rhesus macaques. *Mucosal Immunol.* 9, 1089-1101. 10.1038/mi.2015.127 26647718 PMC4900951

[DMM049256C93] Liu, J., Li, S., Liu, J., Liang, B., Wang, X., Wang, H., Li, W., Tong, Q., Yi, J., Zhao, L. et al. (2020). Longitudinal characteristics of lymphocyte responses and cytokine profiles in the peripheral blood of SARS-CoV-2 infected patients. *EBioMedicine* 55, 102763. 10.1016/j.ebiom.2020.102763 32361250 PMC7165294

[DMM049256C94] Luther, S. A., Ansel, K. M. and Cyster, J. G. (2003). Overlapping roles of CXCL13, interleukin 7 receptor α, and CCR7 ligands in lymph node development. *J. Exp. Med.* 197, 1191-1198. 10.1084/jem.20021294 12732660 PMC2193976

[DMM049256C95] Martinez, V. G., Pankova, V., Krasny, L., Singh, T., Makris, S., White, I. J., Benjamin, A. C., Dertschnig, S., Horsnell, H. L., Kriston-Vizi, J. et al. (2019). Fibroblastic reticular cells control conduit matrix deposition during lymph node expansion. *Cell Reports* 29, 2810-2822.e5. 10.1016/j.celrep.2019.10.103 31775047 PMC6899512

[DMM049256C96] Masters, A. R., Hall, A., Bartley, J. M., Keilich, S. R., Lorenzo, E. C., Jellison, E. R., Puddington, L. and Haynes, L. (2019). Assessment of lymph node stromal cells as an underlying factor in age-related immune impairment. *J. Gerontol. A Biol. Sci. Med. Sci.* 74, 1734-1743. 10.1093/gerona/glz029 30721932 PMC6777091

[DMM049256C97] Matzinger, P. (2002). The danger model: a renewed sense of self. *Science* 296, 301-305. 10.1126/science.1071059 11951032

[DMM049256C98] McElroy, A. K., Akondy, R. S., Davis, C. W., Ellebedy, A. H., Mehta, A. K., Kraft, C. S., Lyon, G. M., Ribner, B. S., Varkey, J., Sidney, J. et al. (2015). Human Ebola virus infection results in substantial immune activation. *Proc. Natl. Acad. Sci. U.S.A.* 112, 4719-4724. 10.1073/pnas.1502619112 25775592 PMC4403189

[DMM049256C99] Meier, F., Will, S., Ellwanger, U., Schlagenhauff, B., Schittek, B., Rassner, G. and Garbe, C. (2002). Metastatic pathways and time courses in the orderly progression of cutaneous melanoma. *Br. J. Dermatol.* 147, 62-70. 10.1046/j.1365-2133.2002.04867.x 12100186

[DMM049256C100] Miller, M. J., Wei, S. H., Parker, I. and Cahalan, M. D. (2002). Two-photon imaging of lymphocyte motility and antigen response in intact lymph node. *Science* 296, 1869-1873. 10.1126/science.1070051 12016203

[DMM049256C101] Mionnet, C., Mondor, I., Jorquera, A., Loosveld, M., Maurizio, J., Arcangeli, M.-L., Ruddle, N. H., Nowak, J., Aurrand-Lions, M., Luche, H. et al. (2013). Identification of a new stromal cell type involved in the regulation of inflamed B cell follicles. *PLoS Biol.* 11, e1001672-e13. 10.1371/journal.pbio.1001672 24130458 PMC3794863

[DMM049256C102] Miteva, D. O., Rutkowski, J. M., Dixon, J. B., Kilarski, W., Shields, J. D. and Swartz, M. A. (2010). Transmural flow modulates cell and fluid transport functions of lymphatic endothelium. *Circ. Res.* 106, 920-931. 10.1161/circresaha.109.207274 20133901 PMC10994404

[DMM049256C103] Mitsdoerffer, M. and Peters, A. (2016). Tertiary lmphoid organs in central nervous system autoimmunity. *Front. Immunol.* 7, 451. 10.3389/fimmu.2016.00451 27826298 PMC5078318

[DMM049256C104] Mondor, I., Baratin, M., Lagueyrie, M., Saro, L., Henri, S., Gentek, R., Suerinck, D., Kastenmüller, W., Jiang, J. X. and Bajénoff, M. (2019). Lymphatic endothelial cells are essential components of the subcapsular sinus macrophage niche. *Immunity* 50, 1453-1466.e4. 10.1016/j.immuni.2019.04.002 31053503 PMC6697131

[DMM049256C105] Monteran, L. and Erez, N. (2019). The dark side of fibroblasts: Cancer-associated fibroblasts as mediators of immunosuppression in the tumor microenvironment. *Front. Immunol.* 10, 1835. 10.3389/fimmu.2019.01835 31428105 PMC6688105

[DMM049256C106] Morris, D. E., Cleary, D. W. and Clarke, S. C. (2017). Secondary bacterial infections associated with influenza pandemics. *Front. Microbiol.* 8, 1041. 10.3389/fmicb.2017.01041 28690590 PMC5481322

[DMM049256C107] Mueller, C. G. and Hess, E. (2012). Emerging functions of RANKL in lymphoid tissues. *Front. Immunol.* 3, 261. 10.3389/fimmu.2012.00261 22969763 PMC3432452

[DMM049256C108] Müller, A., Homey, B., Soto, H., Ge, N., Catron, D., Buchanan, M. E., McClanahan, T., Murphy, E., Yuan, W., Wagner, S. N. et al. (2001). Involvement of chemokine receptors in breast cancer metastasis. *Nature* 410, 50-56. 10.1038/35065016 11242036

[DMM049256C109] Munn, D. H. and Mellor, A. L. (2006). The tumor-draining lymph node as an immune-privileged site. *Immunol. Rev.* 213, 146-158. 10.1111/j.1600-065x.2006.00444.x 16972902

[DMM049256C110] Muñoz-Fontela, C. and McElroy, A. K. (2017). Ebola virus disease in humans: pathophysiology and immunity. In *Marburg- and Ebolaviruses. Current Topics in Microbiology and Immunology*, vol. 411 (ed. E. Mühlberger, L. Hensley, J. Towner), pp. 141-169. Springer Cham. 10.1007/82_2017_11 PMC7122202 28653186

[DMM049256C111] Norkin, M. and Wingard, J. R. (2017). Recent advances in hematopoietic stem cell transplantation. *F1000Res.* 6, 870. 10.12688/f1000research.11233.1 28663793 PMC5473408

[DMM049256C112] Nourshargh, S., Hordijk, P. L. and Sixt, M. (2010). Breaching multiple barriers: leukocyte motility through venular walls and the interstitium. *Nat. Rev. Mol. Cell Biol.* 11, 366-378. 10.1038/nrm2889 20414258

[DMM049256C113] Novkovic, M., Onder, L., Cupovic, J., Abe, J., Bomze, D., Cremasco, V., Scandella, E., Stein, J. V., Bocharov, G., Turley, S. J. et al. (2016). Topological small-world organization of the fibroblastic reticular cell network determines lymph node functionality. *PLoS Biol.* 14, e1002515-e20. 10.1371/journal.pbio.1002515 27415420 PMC4945005

[DMM049256C114] Novkovic, M., Onder, L., Bocharov, G. and Ludewig, B. (2020). Topological structure and robustness of the lymph node conduit system. *Cell Reports* 30, 893-904.e6. 10.1016/j.celrep.2019.12.070 31968261

[DMM049256C115] Núñez, N. G., Boari, J. T., Ramos, R. N., Richer, W., Cagnard, N., Anderfuhren, C. D., Niborski, L. L., Bigot, J., Meseure, D., Rochere, P. et al. (2020). Tumor invasion in draining lymph nodes is associated with Treg accumulation in breast cancer patients. *Nat. Commun.* 11, 3272. 10.1038/s41467-020-17046-2 32601304 PMC7324591

[DMM049256C116] Onder, L., Mörbe, U., Pikor, N., Novkovic, M., Cheng, H.-W., Hehlgans, T., Pfeffer, K., Becher, B., Waisman, A., Rülicke, T. et al. (2017). Lymphatic endothelial cells control initiation of lymph node organogenesis. *Immunity* 47, 80-92.e4. 10.1016/j.immuni.2017.05.008 28709801

[DMM049256C117] Pan, F., Zheng, C., Ye, T., Li, L., Liu, D., Li, L., Hesketh, R. L. and Yang, L. (2020). Different computed tomography patterns of Coronavirus Disease 2019 (COVID-19) between survivors and non-survivors. *Sci. Rep.* 10, 11336. 10.1038/s41598-020-68057-4 32647307 PMC7347874

[DMM049256C118] Pelon, F., Bourachot, B., Kieffer, Y., Magagna, I., Mermet-Meillon, F., Bonnet, I., Costa, A., Givel, A.-M., Attieh, Y., Barbazan, J. et al. (2020). Cancer-associated fibroblast heterogeneity in axillary lymph nodes drives metastases in breast cancer through complementary mechanisms. *Nat. Commun.* 11, 404-420. 10.1038/s41467-019-14134-w 31964880 PMC6972713

[DMM049256C119] Perez-Shibayama, C., Gil-Cruz, C. and Ludewig, B. (2019). Fibroblastic reticular cells at the nexus of innate and adaptive immune responses. *Immunol. Rev.* 289, 31-41. 10.1111/imr.12748 30977192 PMC6850313

[DMM049256C120] Perkey, E. and Maillard, I. (2018). New insights into graft-versus-host disease and graft rejection. *Annu. Rev. of Pathol.* 13, 219-245. 10.1146/annurev-pathol-020117-043720 29099650

[DMM049256C121] Peters, A., Pitcher, L. A., Sullivan, J. M., Mitsdoerffer, M., Acton, S. E., Franz, B., Wucherpfennig, K., Turley, S., Carroll, M. C., Sobel, R. A. et al. (2011). Th17 cells induce ectopic lymphoid follicles in central nervous system tissue inflammation. *Immunity* 35, 986-996. 10.1016/j.immuni.2011.10.015 22177922 PMC3422678

[DMM049256C122] Pikor, N. B., Astarita, J. L., Summers-Deluca, L., Galicia, G., Qu, J., Ward, L. A., Armstrong, S., Dominguez, C. X., Malhotra, D., Heiden, B. et al. (2015a). Integration of Th17- and lymphotoxin-derived signals initiates meningeal-resident stromal cell remodeling to propagate neuroinflammation. *Immunity* 43, 1160-1173. 10.1016/j.immuni.2015.11.010 26682987

[DMM049256C123] Pikor, N. B., Prat, A., Bar-Or, A. and Gommerman, J. L. (2015b). Meningeal tertiary lymphoid tissues and multiple sclerosis: A gathering place for diverse types of immune cells during CNS autoimmunity. *Front. Immunol.* 6, 657. 10.3389/fimmu.2015.00657 26793195 PMC4710700

[DMM049256C124] Pipi, E., Nayar, S., Gardner, D. H., Colafrancesco, S., Smith, C. and Barone, F. (2018). Tertiary lymphoid structures: Autoimmunity goes local. *Front. Immunol.* 9, 1952. 10.3389/fimmu.2018.01952 30258435 PMC6143705

[DMM049256C125] Pociask, D. A., Scheller, E. V., Mandalapu, S., McHugh, K. J., Enelow, R. I., Fattman, C. L., Kolls, J. K. and Alcorn, J. F. (2013). IL-22 is essential for lung epithelial repair following influenza infection. *Am. J. Pathol.* 182, 1286-1296. 10.1016/j.ajpath.2012.12.007 23490254 PMC3620404

[DMM049256C126] Polak, S. B., Gool, I. C. V., Cohen, D., von der Thüsen, J. H. and van Paassen, J. (2020). A systematic review of pathological findings in COVID-19: a pathophysiological timeline and possible mechanisms of disease progression. *Mod. Pathol.* 33, 2128-2138. 10.1038/s41379-020-0603-3 32572155 PMC7306927

[DMM049256C127] Prescott, J. B., Marzi, A., Safronetz, D., Robertson, S. J., Feldmann, H. and Best, S. M. (2017). Immunobiology of Ebola and Lassa virus infections. *Nat. Rev. Immunol.* 17, 195-207. 10.1038/nri.2016.138 28111475

[DMM049256C128] Rangel-Moreno, J., Carragher, D. M., de la Luz Garcia, -Hernandez, M., Hwang, J. Y., Kusser, K., Hartson, L., Kolls, J. K., Khader, S. A. and Randall, T. D. (2011). The development of inducible bronchus associated lymphoid tissue (iBALT) is dependent on IL-17. *Nat. Immunol.* 12, 639-646. 10.1038/ni.2053 21666689 PMC3520063

[DMM049256C129] Reichenbach, D. K., Schwarze, V., Matta, B. M., Tkachev, V., Lieberknecht, E., Liu, Q., Koehn, B. H., Pfeifer, D., Taylor, P. A., Prinz, G. et al. (2015). The IL-33/ST2 axis augments effector T-cell responses during acute GVHD. *Blood* 125, 3183-3192. 10.1182/blood-2014-10-606830 25814531 PMC4432012

[DMM049256C130] Riedel, A., Shorthouse, D., Haas, L., Hall, B. A. and Shields, J. (2016). Tumor-induced stromal reprogramming drives lymph node transformation. *Nat. Immunol.* 17, 1118-1127. 10.1038/ni.3492 27400148 PMC4994871

[DMM049256C131] Rodda, L. B., Lu, E., Bennett, M. L., Sokol, C. L., Wang, X., Luther, S. A., Barres, B. A., Luster, A. D., Ye, C. J. and Cyster, J. G. (2018). Single-cell RNA sequencing of lymph node stromal cells reveals niche-associated heterogeneity. *Immunity* 48, 1014-1028.e6. 10.1016/j.immuni.2018.04.006 29752062 PMC5971117

[DMM049256C132] Rodriguez, A. B., Peske, J. D., Woods, A. N., Leick, K. M., Mauldin, I. S., Meneveau, M. O., Young, S. J., Lindsay, R. S., Melssen, M. M., Cyranowski, S. et al. (2021). Immune mechanisms orchestrate tertiary lymphoid structures in tumors via cancer-associated fibroblasts. *Cell Reports* 36, 109422. 10.1016/j.celrep.2021.109422 34289373 PMC8362934

[DMM049256C133] Ruddle, N. H. and Akirav, E. M. (2009). Secondary lymphoid organs: responding to genetic and environmental cues in ontogeny and the immune response. *J. Immunol.* 183, 2205-2212. 10.4049/jimmunol.0804324 19661265 PMC2766168

[DMM049256C134] Sahai, E., Astsaturov, I., Cukierman, E., DeNardo, D. G., Egeblad, M., Evans, R. M., Fearon, D., Greten, F. R., Hingorani, S. R., Hunter, T. et al. (2020). A framework for advancing our understanding of cancer-associated fibroblasts. *Nat. Rev. Cancer* 20, 174-186. 10.1038/s41568-019-0238-1 31980749 PMC7046529

[DMM049256C135] Sandy, A. R., Chung, J., Toubai, T., Shan, G. T., Tran, I. T., Friedman, A., Blackwell, T. S., Reddy, P., King, P. D. and Maillard, I. (2013). T cell-specific notch inhibition blocks graft-versus-host disease by inducing a hyporesponsive program in alloreactive CD^4+^ and CD^8+^ T cells. *J. Immunol.* 190, 5818-5828. 10.4049/jimmunol.1203452 23636056 PMC3660433

[DMM049256C136] Santi, P. A. (2011). Light sheet fluorescence microscopy: a review. *J. Histochem. Cytochem.* 59, 129-138. 10.1369/0022155410394857 21339178 PMC3201139

[DMM049256C137] Sanz-Moreno, V., Gaggioli, C., Yeo, M., Albrengues, J., Wallberg, F., Viros, A., Hooper, S., Mitter, R., Féral, C. C., Cook, M. et al. (2011). ROCK and JAK1 signaling cooperate to control actomyosin contractility in tumor cells and stroma. *Cancer Cell* 20, 229-245. 10.1016/j.ccr.2011.06.018 21840487

[DMM049256C138] Shields, J. D., Kourtis, I. C., Tomei, A. A., Roberts, J. M. and Swartz, M. A. (2010). Induction of lymphoidlike stroma and immune escape by tumors that express the chemokine CCL21. *Science* 328, 749-752. 10.1126/science.1185837 20339029

[DMM049256C139] Shono, Y., Docampo, M. D., Peled, J. U., Perobelli, S. M., Velardi, E., Tsai, J. J., Slingerland, A. E., Smith, O. M., Young, L. F., Gupta, J. et al. (2016). Increased GVHD-related mortality with broad-spectrum antibiotic use after allogeneic hematopoietic stem cell transplantation in human patients and mice. *Sci. Transl. Med.* 8, 339ra71. 10.1126/scitranslmed.aaf2311 PMC4991773 27194729

[DMM049256C170] Siegert, S., Huang, H.-Y., Yang, C.-Y., Scarpellino, L., Carrie, L., Essex, S., Nelson, P. J., Heikenwalder, M., Acha-Orbea, H., Buckley, C. D. et al. (2011). Fibroblastic reticular cells from lymph nodes attenuate T cell expansion by producing nitric oxide. *PLoS ONE* 6, e27618. 10.1371/journal.pone.0027618 PMC3215737 22110693

[DMM049256C140] Simic, M., Manosalva, I., Spinelli, L., Gentek, R., Shayan, R. R., Siret, C., Girard-Madoux, M., Wang, S., de Fabritus, L., Verschoor, J. et al. (2020). Distinct waves from the hemogenic endothelium give rise to layered lymphoid tissue inducer cell ontogeny. *Cell Reports* 32, 108004. 10.1016/j.celrep.2020.108004 32783932

[DMM049256C141] Singh, R. and Choi, B. K. (2019). Siglec1-expressing subcapsular sinus macrophages provide soil for melanoma lymph node metastasis. *ELife* 8, 804-819. 10.7554/elife.48916 PMC6930078 31872800

[DMM049256C142] Sixt, M., Kanazawa, N., Selg, M., Samson, T., Roos, G., Reinhardt, D. P., Pabst, R., Lutz, M. B. and Sorokin, L. (2005). The conduit system transports soluble antigens from the afferent lymph to resident dendritic cells in the T cell area of the lymph node. *Immunity* 22, 19-29. 10.1016/j.immuni.2004.11.013 15664156

[DMM049256C143] Steele, K. E., Anderson, A. O. and Mohamadzadeh, M. (2009). Fibroblastic reticular cell infection by hemorrhagic fever viruses. *Immunotherapy* 1, 187-197. 10.2217/1750743x.1.2.187 20635940

[DMM049256C144] Suenaga, F., Ueha, S., Abe, J., Kosugi-Kanaya, M., Wang, Y., Yokoyama, A., Shono, Y., Shand, F. H. W., Morishita, Y., Kunisawa, J. et al. (2014). Loss of lymph node fibroblastic reticular cells and high endothelial cells is associated with humoral immunodeficiency in mouse graft-versus-host disease. *J. Immunol.* 194, 398-406. 10.4049/jimmunol.1401022 25422510

[DMM049256C171] Susaki, E. A., Tainaka, K., Perrin, D., Kishino, F., Tawara, T., Watanabe, T. M., Yokoyama, C., Onoe, H., Eguchi, M., Yamaguchi, S. et al. (2014). Whole-brain imaging with single-cell resolution using chemical cocktails and computational analysis. *Cell* 157, 726-739. 10.1016/j.cell.2014.03.042 24746791

[DMM049256C145] Tang, H., Zhu, M., Qiao, J. and Fu, Y.-X. (2017). Lymphotoxin signalling in tertiary lymphoid structures and immunotherapy. *Cell. Mol. Immunol.* 14, 809-818. 10.1038/cmi.2017.13 28413217 PMC5649108

[DMM049256C146] Thierry, G. R., Kuka, M., Giovanni, M. D., Mondor, I., Brouilly, N., Iannacone, M. and Bajénoff, M. (2018). The conduit system exports locally secreted IgM from lymph nodes. *J. Exp. Med.* 215, 2972-2983. 10.1084/jem.20180344 30429248 PMC6279403

[DMM049256C147] Tsukamoto, H., Chernogorova, P., Ayata, K., Gerlach, U. V., Rughani, A., Ritchey, J. W., Ganesan, J., Follo, M., Zeiser, R., Thompson, L. F. et al. (2012). Deficiency of CD73/ecto-5′-nucleotidase in mice enhances acute graft-versus-host disease. *Blood* 119, 4554-4564. 10.1182/blood-2011-09-375899 22262774 PMC3362368

[DMM049256C148] Twenhafel, N. A., Mattix, M. E., Johnson, J. C., Robinson, C. G., Pratt, W. D., Cashman, K. A., Wahl-Jensen, V., Terry, C., Olinger, G. G., Hensley, L. E. et al. (2013). Pathology of experimental aerosol Zaire ebolavirus infection in rhesus macaques. *Vet. Pathol.* 50, 514-529. 10.1177/0300985812469636 23262834

[DMM049256C149] Ulvmar, M. H., Werth, K., Braun, A., Kelay, P., Hub, E., Eller, K., Chan, L., Lucas, B., Novitzky-Basso, I., Nakamura, K. et al. (2014). The atypical chemokine receptor CCRL1 shapes functional CCL21 gradients in lymph nodes. *Nat. Immunol.* 15, 623-630. 10.1038/ni.2889 24813163

[DMM049256C150] Van den Eynden, G. G., Van der Auwera, I., Van Laere,, S. J., Huygelen, V., Colpaert, C. G., Van Dam, P., Dirix, L. Y., Vermeulen, P. B. and Van Marck, E. A. (2006). Induction of lymphangiogenesis in and around axillary lymph node metastases of patients with breast cancer. *Br. J. Cancer* 95, 1362-1366. 10.1038/sj.bjc.6603443 17088912 PMC2360596

[DMM049256C151] Verdoni, L., Mazza, A., Gervasoni, A., Martelli, L., Ruggeri, M., Ciuffreda, M., Bonanomi, E. and D'Antiga, L. (2020). An outbreak of severe Kawasaki-like disease at the Italian epicentre of the SARS-CoV-2 epidemic: an observational cohort study. *Lancet* 395, 1771-1778. 10.1016/s0140-6736(20)31103-x 32410760 PMC7220177

[DMM049256C152] Vezys, V., Olson, S. and Lefrançois, L. (2000). Expression of intestine-specific antigen reveals novel pathways of CD8 T cell tolerance induction. *Immunity* 12, 505-514. 10.1016/s1074-7613(00)80202-2 10843383

[DMM049256C169] Wang, W., Gong, F., Zhu, W., Fu, S. and Zhang, Q. (2015). Macrophage activation syndrome in Kawasaki disease: more common than we thought? *Semin. Arthritis Rheum*. 44, 405-410. 10.1016/j.semarthrit.2014.07.007 25200945

[DMM049256C153] Wang, X.-X., Shao, C., Huang, X.-J., Sun, L., Meng, L.-J., Liu, H., Zhang, S.-J., Li, H.-J. and Lv, F.-D. (2021). Histopathological features of multiorgan percutaneous tissue core biopsy in patients with COVID-19. *J. Clin. Pathol.* 74, 522-527. 10.1136/jclinpath-2020-206623 32848014 PMC8311110

[DMM049256C154] Wauquier, N., Becquart, P., Padilla, C., Baize, S. and Leroy, E. M. (2010). Human fatal zaire ebola virus infection is associated with an aberrant innate immunity and with massive lymphocyte apoptosis. *PLoS Negl. Trop. Dis.* 4, e837. 10.1371/journal.pntd.0000837 20957152 PMC2950153

[DMM049256C155] Wilhelm, K., Ganesan, J., Müller, T., Dürr, C., Grimm, M., Beilhack, A., Krempl, C. D., Sorichter, S., Gerlach, U. V., Jüttner, E. et al. (2010). Graft-versus-host disease is enhanced by extracellular ATP activating P2X7R. *Nat. Med.* 16, 1434-1438. 10.1038/nm.2242 21102458

[DMM049256C156] Wood, S., Feng, J., Chung, J., Radojcic, V., Sandy-Sloat, A. R., Friedman, A., Shelton, A., Yan, M., Siebel, C. W., Bishop, D. K. et al. (2015). Transient blockade of delta-like Notch ligands prevents allograft rejection mediated by cellular and humoral mechanisms in a mouse model of heart transplantation. *J. Immunol.* 194, 2899-2908. 10.4049/jimmunol.1402034 25687759 PMC4355388

[DMM049256C157] Wu, J., Liang, Y., Chen, S., Hsu, C.-L., Chavarha, M., Evans, S. W., Shi, D., Lin, M. Z., Tsia, K. K. and Ji, N. (2020). Kilohertz two-photon fluorescence microscopy imaging of neural activity in vivo. *Nat. Methods* 17, 287-290. 10.1038/s41592-020-0762-7 32123392 PMC7199528

[DMM049256C158] Xiang, M., Grosso, R. A., Takeda, A., Pan, J., Bekkhus, T., Brulois, K., Dermadi, D., Nordling, S., Vanlandewijck, M., Jalkanen, S. et al. (2020). A single-cell transcriptional roadmap of the mouse and human lymph node lymphatic vasculature. *Front. Cardiovasc. Med.* 7, 52. 10.3389/fcvm.2020.00052 32426372 PMC7204639

[DMM049256C159] Yang, B., Treweek, J. B., Kulkarni, R. P., Deverman, B. E., Chen, C.-K., Lubeck, E., Shah, S., Cai, L. and Gradinaru, V. (2014). Single-cell phenotyping within transparent intact tissue through whole-body clearing. *Cell* 158, 945-958. 10.1016/j.cell.2014.07.017 25088144 PMC4153367

[DMM049256C160] Yip, L., Su, L., Sheng, D., Chang, P., Atkinson, M., Czesak, M., Albert, P. R., Collier, A.-R., Turley, S. J., Fathman, C. G. et al. (2009). Deaf1 isoforms control the expression of genes encoding peripheral tissue antigens in the pancreatic lymph nodes during type 1 diabetes. *Nat. Immunol.* 10, 1026-1033. 10.1038/ni.1773 19668219 PMC2752139

[DMM049256C161] Yu, F.-X., Zhao, B. and Guan, K.-L. (2015). Hippo pathway in organ size control, tissue homeostasis, and cancer. *Cell* 163, 811-828. 10.1016/j.cell.2015.10.044 26544935 PMC4638384

[DMM049256C162] Zeiser, R. and Blazar, B. R. (2017). Pathophysiology of chronic graft-versus-host disease and therapeutic targets. *N. Engl. J. Med.* 377, 2565-2579. 10.1056/nejmra1703472 29281578

[DMM049256C163] Zelenay, S. and Sousa, C. R. E. (2013). Adaptive immunity after cell death. *Trends Immunol.* 34, 329-335. 10.1016/j.it.2013.03.005 23608152

[DMM049256C164] Zhang, G. J., Tsuda, H., Adachi, I., Fukutomi, T., Yamamoto, H. and Hirohashi, S. (1997). Prognostic indicators for breast cancer patients with one to three regional lymph node metastases, with special reference to alterations in expression levels of bcl-2, p53 and c-erbB-2 proteins. *Jpn. J. Clin. Oncol.* 27, 371-377. 10.1093/jjco/27.6.371 9437997

[DMM049256C165] Zhang, J., Ramadan, A. M., Griesenauer, B., Li, W., Turner, M. J., Liu, C., Kapur, R., Hanenberg, H., Blazar, B. R., Tawara, I. et al. (2015). ST2 blockade reduces sST2-producing T cells while maintaining protective mST2-expressing T cells during graft-versus-host disease. *Sci. Transl. Med.* 7, 308ra160. 10.1126/scitranslmed.aab0166 PMC4699312 26446957

[DMM049256C166] Zhou, F., Yu, T., Du, R., Fan, G., Liu, Y., Liu, Z., Xiang, J., Wang, Y., Song, B., Gu, X. et al. (2020). Clinical course and risk factors for mortality of adult inpatients with COVID-19 in Wuhan, China: a retrospective cohort study.*Lancet* 395, 1054-1062. 10.1016/s0140-6736(20)30566-3 32171076 PMC7270627

[DMM049256C167] Ziegler, C. G. K., Allon, S. J., Nyquist, S. K., Mbano, I. M., Miao, V. N., Tzouanas, C. N., Cao, Y., Yousif, A. S., Bals, J., Hauser, B. M. et al. (2020). SARS-CoV-2 receptor ACE2 is an interferon-stimulated gene in human airway epithelial cells and is detected in specific cell subsets across tissues. *Cell* 181, 1016-1035.e19. 10.1016/j.cell.2020.04.035 32413319 PMC7252096

